# Oxidative addition of carbon–fluorine and carbon–oxygen bonds to Al(i)†
†Electronic supplementary information (ESI) available: Including experimental procedures, X-ray crystallography data and characterisation data. CCDC 1419650-1419651. For ESI and crystallographic data in CIF or other electronic format see DOI: 10.1039/c5cc07140b



**DOI:** 10.1039/c5cc07140b

**Published:** 2015-09-21

**Authors:** Mark R. Crimmin, Michael J. Butler, Andrew J. P. White

**Affiliations:** a Department of Chemistry , Imperial College London , South Kensington , London SW7 2AZ , UK . Email: m.crimmin@imperial.ac.uk ; Tel: +44 (0)2075942846

## Abstract

Addition of fluoroarenes, fluoroalkanes or benzofuran to [{(2,6-^i^Pr_2_C_6_H_3_NCMe)_2_CH}Al] results in facile oxidative addition of either a C–F or C–O bond to the Al(i) centre.

## Abstract 2

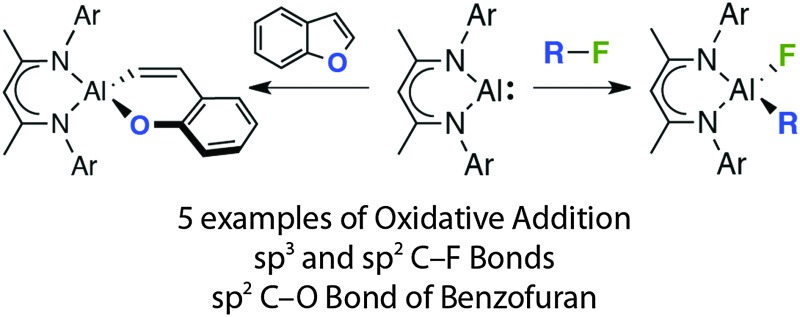
Addition of fluoroarenes, fluoroalkanes or benzofuran to [{(2,6-^i^Pr_2_C_6_H_3_NCMe)_2_CH}Al] results in facile oxidative addition of either a C–F or C–O bond to the Al(i) centre.

The addition of small molecules to ‘heavy carbenes’ of group 14 has led to the discovery of some remarkable reactivity.^1^ For example, ligand stabilised silylene, germylene and stannylene complexes react non-reversibly with fluorinated arenes through either carbon–fluorine or carbon–hydrogen bond functionalisation.^2^ In related studies of group 13 analogues, an isolable monomeric Al(i) complex has been reported to undergo oxidative addition of substrates containing H–H, O–H, N–H, P–H, acidic C–H, H–Si, H–B, H–Al, Bi–Bi and Sb–Sb bonds.^3,4^

For our part, we have shown that in the presence of catalytic quantities of [Cp_2_ZrCl_2_] or [Cp*RhCl(μ-Cl)]_2_, the aluminium dihydride **1** can be applied as a stoichiometric reagent for carbon–fluorine and carbon–oxygen bond cleavage in fluorocarbons and benzofuran (Fig. 1).^5,6^ In the case of fluorocarbons two competing pathways are observed: hydrodefluorination and C–F alumination.^5^

**Fig. 1:**
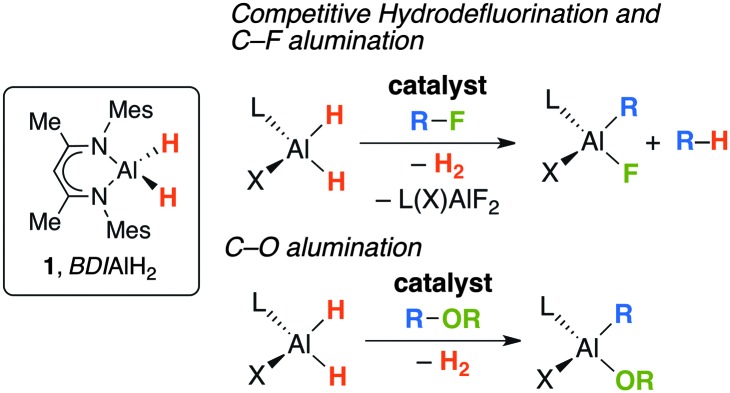
Transition metal catalysed R–F and R–OR bond functionalisation with the aluminium dihydride **1**.

One possible mechanism for R–X (X = F, OR) bond cleavage is through a transition-metal catalysed dehydrogenation of **1** to form an Al(i) intermediate.^7^ This low-valent intermediate could then effect carbon–fluorine or carbon–oxygen bond cleavage by oxidative addition. In the case of the fluoroarenes a competitive hydride transfer pathway could explain the hydrodefluorination products. While this hypothesis is just one of a number of possible mechanisms for R–X bond activation, two points lend weight to a hypothetical Al(iii) → Al(i) transformation under catalytic conditions. (i) A recent detailed analysis of the electronic structure of **2** (Fig. 2) and the analogous Al(iii) dihydride has exposed striking similarities between the charge density at aluminium in these complexes as evidenced by a combination of polarized aluminium K-edge X-ray absorption near edge structure (XANES) spectroscopy and calculations.^8^ (ii) While exploring the coordination chemistry of **1** we,^9^ and others,^10^ have shown that on-metal dehydrogenation is possible. Despite the rich bond activation chemistry of carbene analogues of the main group, the addition of R–X bonds to Al(i) has limited precedent.^11^ In this communication, we show that oxidative addition of C–F and C–O bonds to Al(i) is facile (Fig. 2). The findings mean that we cannot, as yet, discount the involvement of low-valent aluminium intermediates in the previous catalytic studies (Fig. 1).

**Fig. 2:**
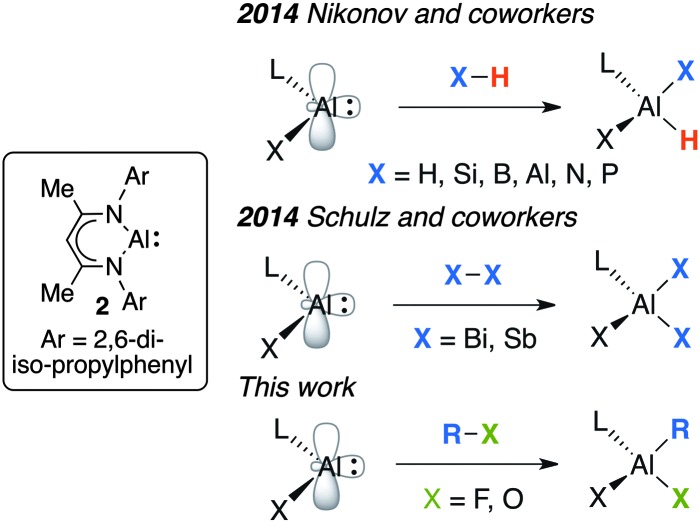
Oxidative addition of X–H, X–X and R–X bonds to **2**.

The reaction of **2** with an excess of hexafluorobenzene or pentafluorobenzene in C_6_D_6_ proceeded rapidly at room temperature. Upon addition of the fluoroarene to a solution of **2** an instant colour change was observed from red/orange to a pale yellow. Following reactions by ^1^H and ^19^F NMR spectroscopy revealed the clean formation of **3a–b** derived from the oxidative addition of an sp^2^ C–F bond to **2** (Scheme 1).

**Scheme 1:**
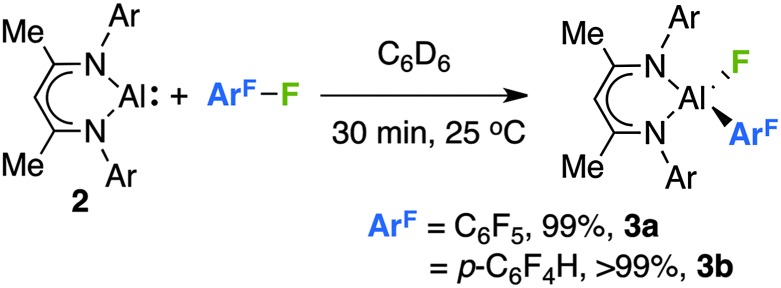
The reaction of **2** with fluoroarenes, yields are recorded by ^1^H NMR by comparison to ferrocene as an internal standard.

No additional species were observed in solution and no evidence for competitive hydrodefluorination was obtained. For pentafluorobenzene, C–F bond cleavage occurs *para* to the existing carbon–hydrogen bond to give a product containing a 2,3,5,6-tetrafluoroarene motif. The regiochemistry in **3b** was established through comparison of ^19^F NMR data with our previous findings,^5^ and **3b** demonstrates ^19^F resonances at *δ* = –120.5 (m, *ortho*), –139.8 (m, *meta*) and –168 (broad s, Al–F) ppm. It is worth noting that the silylene analogue of **2** reacts with pentafluorobenzene by oxidative addition of a C–H bond.^2*a*^ While reactions of **2** with 1,2,3-trifluorobenzene or fluorobenzene at 25–80 °C gave evidence for the slow formation of new aluminium fluoride species over 1–2 weeks, neither reaction cleanly produced analogues of **3a**/**b**.

The scope of this transformation is not limited to the sp^2^ C–F bonds of fluoroarenes. Addition of 1-fluorohexane or fluorocyclohexane to **2** in C_6_D_6_ yielded the corresponding aluminium alkyls **3c–d** through cleavage of the sp^3^ C–F bond (Scheme 2). In these instances, in addition to the expected aluminium fluoride resonances in ^19^F NMR spectra (**3c**, –161.8 ppm; **3d**, –157.7 ppm) diagnostic aluminium alkyl resonances were observed between –0.2 and +0.3 ppm in ^1^H NMR spectra.

**Scheme 2:**
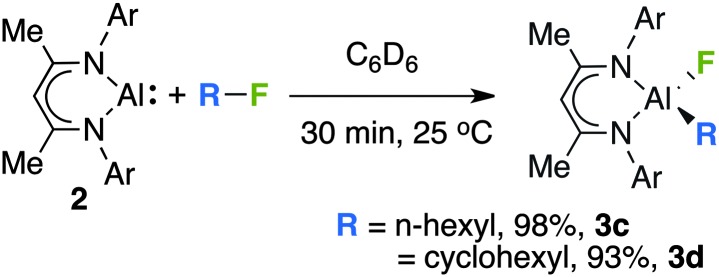
The reaction of **2** with fluoroalkanes, yields are recorded by ^1^H NMR by comparison to ferrocene as an internal standard.

Although **2** did not react with benzofuran at 25 °C in C_6_D_6_, upon heating to 80 °C slow conversion to **4** was observed over a period of 24 h (Scheme 3). Diagnostic resonances of the aluminium vinyl unit of the product were observed at *δ* = 5.99 (d, ^3^ *J*_H–H_ = 16.0 Hz) and 7.44 (d, ^3^ *J*_H–H_ = 16.0 Hz) ppm. In contrast to the zirconocene dichloride catalysed reaction of **1** with benzofuran, no support for the formation of a saturated analogue of **4** was obtained.^5^ The reaction is selective for the formation of a 6-membered metallocycle containing a *Z*-alkenyl unit from oxidative addition of the sp^2^ C–O bond of benzofuran to Al(i).

**Scheme 3:**
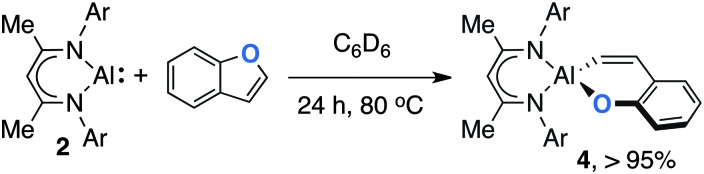
The reaction of **2** with benzofuran, yield is recorded by ^1^H NMR by comparison to ferrocene as an internal standard.

The organometallic products of these reactions have been characterised by multinuclear NMR spectroscopy and in the case of **3a** and **4** by single crystal X-ray diffraction (Fig. 3). The aluminium–carbon and aluminium–fluorine bond lengths in the three independent complexes in **3a** take values of 1.9916(19)–2.0020(18) and 1.6582(11)–1.6619(11) Å respectively. These data are consistent with those found in [BDIAl(C_6_F_5_)_2_] (1.9946(15) and 2.0198(15) Å) and [BDIAl(F)_2_] (1.6637(8) and 1.6647(8) Å).^5^ The crystal structure of an analogue of **4** has been previously reported.^6^

**Fig. 3:**
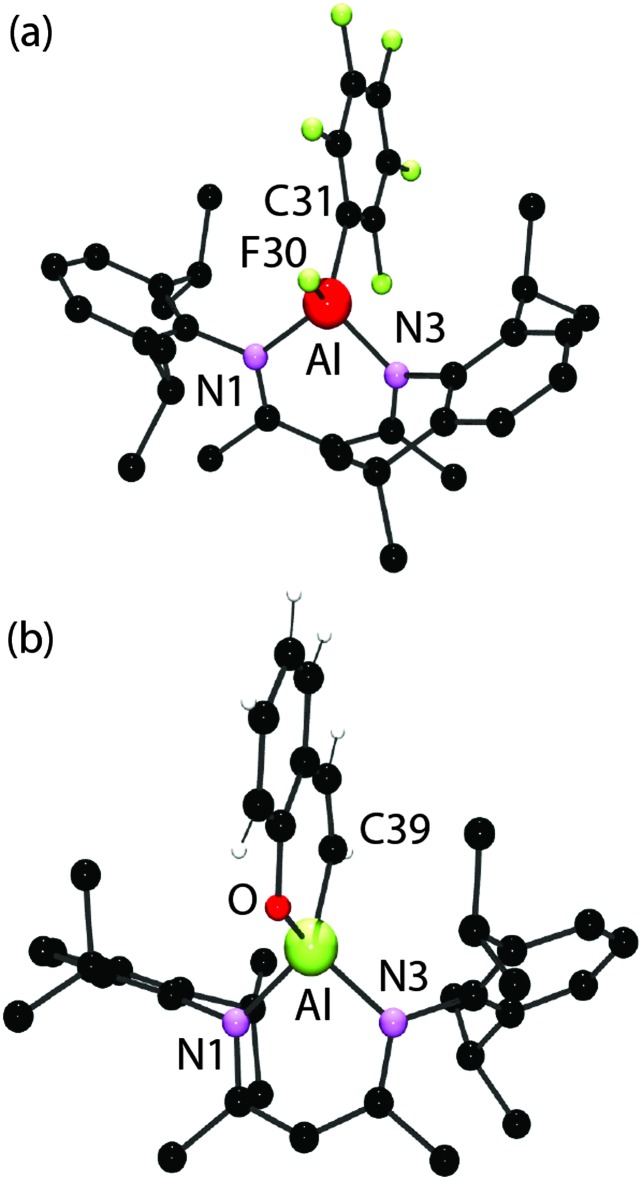
(a) The structure of one (3a-A) of the three independent complexes present in the crystal of **3a**. (b) The crystal structure of **4**. Selected bond lengths (Å) and angles (°): **3** Al(a)–N1(a) 1.8809(14), Al(a)–N3(a) 1.8671(15), Al(a)–F30(a) 1.6582(11), Al(a)–C31(a) 1.9993(18), F30(a)–Al(a)–C31(a) 109.68(7), N1(a)–Al(a)–N3(a) 98.85(6). **4** Al–N1 1.885(2), Al–N3 1.890(2), Al–O31 1.7437(19), Al–C39 1.955(3), O31–Al–C39 102.18(11), N1–Al–N3 97.92(9).

In summary, we have reported the facile oxidative addition of R–X (X = F, OR) bonds in fluoroarenes, fluoroalkanes and benzofuran to the Al(i) complex **2**. From a mechanistic perspective, these data highlight the possibility that an *in situ* transition metal catalysed Al(iii) → Al(i) transformation may be operating in our previously reported bond functionalisation chemistry employing the Al(iii) dihydride **1**.^5,6^ From a more pragmatic point of view, the reactions result in the generation of a new carbon–aluminium bond from ‘inert’ carbon–fluorine or carbon–oxygen bonds. This approach represents a method for directly generating reactive intermediates from non-reactive chemical feedstocks, that uses the most abundant metal in the earth's crust without the need for expensive or toxic transition metal catalysts.^12,13^

We are grateful to the Royal Society (MRC) and EPSRC (EP/L011514/1) for funding. Olga Ekkert is thanked for helpful discussions.

## References

[cit1] Haff M., Schmedake T. A., West R. (2000). Acc. Chem. Res..

[cit2] Jana A., Samuel P. P., Tavcar G., Roesky H. W., Schulzke C. (2010). J. Am. Chem. Soc..

[cit3] Chu T., Korobkov I., Nikonov G. I. (2014). J. Am. Chem. Soc..

[cit4] Ganesamoorthy C., Bläser D., Wölper C., Schulz S. (2014). Angew. Chem., Int. Ed..

[cit5] Yow S., Gates S. J., White A. J. P., Crimmin M. R. (2012). Angew. Chem., Int. Ed..

[cit6] Yow S., Nako A. E., Neveu L., White A. J. P., Crimmin M. R. (2013). Organometallics.

[cit7] Cui C., Roesky H. W., Schmidt H.-G., Noltemeyer M., Hao H., Cimpoesu F. (2000). Angew. Chem., Int. Ed..

[cit8] Altman A. B., Pemmaraju C. D., Camp C., Arnold J., Minasian S. G., Prendergast D., Shuh D. K., Tyliszczak T. (2015). J. Am. Chem. Soc..

[cit9] Ekkert O., White A. J. P., Toms H., Crimmin M. R. (2015). Chem. Sci..

[cit10] Riddlestone I. M., Edmonds S., Kaufman P. A., Urbano J., Bates J. I., Kelly M. J., Thompson A. L., Taylor R., Aldridge S. (2012). J. Am. Chem. Soc..

[cit11] For an example of a C–O bond cleavage product formed directly from the reduction of an Al(iii) precursor in THF see: Schnitter C. Roesky H. W. Röpken C. Herbst-Irmer R. Schmidt H.-G. Noltemeyer M., Angew. Chem., Int. Ed., 1998, 37 , 1952 .

[cit12] Ishii Y., Chatani N., Yorimitsu S., Murai S. (1998). Chem. Lett..

[cit13] Kinuta H., Tobisu M., Chatani N. (2015). J. Am. Chem. Soc..

