# Supplementary material for: Oxidative addition of carbon–fluorine and carbon–oxygen bonds to Al(i)
Source: Chem Commun (Camb). 2015 Sep 21;51(88):15994–6. doi: 10.1039/c5cc07140b (PMC4621529; doi:10.1039/c5cc07140b)
Supplement: Supplementary file 1 [file CC-051-C5CC07140B-s001.pdf]

Electronic Supplementary Material (ESI) for ChemComm.
This journal is © The Royal Society of Chemistry 2015

Supporting Information for:

Oxidative Addition of Carbon–Fluorine and Carbon–Oxygen Bonds to Al(I)

Mark R. Crimmin, Michael J. Butler and Andrew J. P. White

Department of Chemistry, Imperial College London, South Kensington, London SW7 2AZ, U.K. Tel: +44(0)2075942846; E-mail: m.crimmin@imperial.ac.uk

General Experimental

All manipulations were carried out under standard Schlenk-line or glovebox techniques under an inert atmosphere of nitrogen. A MBRAUN Labmaster glovebox was employed operating under an inert atmosphere of concentrations of H_2O and O_2 below 0.1 ppm. Glassware was dried for 12 hours at 120 °C prior to use. C_6D_6 and D_8-toluene were stored over molecular sieves for twelve hours before using. All other solvents were dried using a Grubbs type solvent purification system. ^{1}H, ^{13}C, ^{29}Si NMR experiments were run within Youngs Tap NMR tubes on 400 MHz or 500 MHz BRUCKER machines. HSQC, and DEPT-135 experiments were run on a 400 MHz or 500 MHz NMR machine. The majority of ^{1}H spectra were recorded from + 30ppm to - 20ppm. Spectra were referenced to known solvent peaks. NMR analysis was conducted in Topspin or MestReNova with baseline corrections applied to spectra. All attempts to acquire satisfactory elemental analysis failed. Complex **2** was synthesised according to the literature procedure.[1]

Synthetic Procedure for Oxidative Addition to Al(I)

Procedure for the addition of fluoroarenes, fluoroalkanes (pentafluorobenzene, cyclohexylfluoride, n-hexylfluoride, hexafluorobenzene) to **2:**In a glovebox **2** (20 mg, 0.045 mmol) was weighed into a 2mL scintillation vial and dissolved in 0.5 mL of benzene-d6, ferrocene (1-2 mg) was added to the solution and the mixture transferred to a J Youngs NMR tube. An initial time point was measured by ^{1}H NMR spectroscopy and the mixture returned to the glovebox, the fluorohydrocarbon or benzofuran (0.225 mmol, 5 equiv.) was added by micropipette For the fluorocarbons, an immediate colour change from red to yellow was observed. The reactions were monitorred by ^{1}H and ^{19}F NMR spectroscopy and showed formation of the corresponding products **3a-d** after 30 min at 25 ^{o}C. For benzofuran the mixture was heated to 80 ^{o}C for 24h and slow conversion to **4** was observed. At the end point of the reaction, the products were isolated by removal of the solvent and recrystallisation from n-hexane. NMR yields were measured by comparison to the ferrocene internal standard.

1 [1] C. Cui, H. W. Roesky, H.-G. Schmidt, M. Noltemeyer, H. Hao, F. Cimpoesu, *Angew. Chem., Int. Ed.* 2000, **39**, 4274.

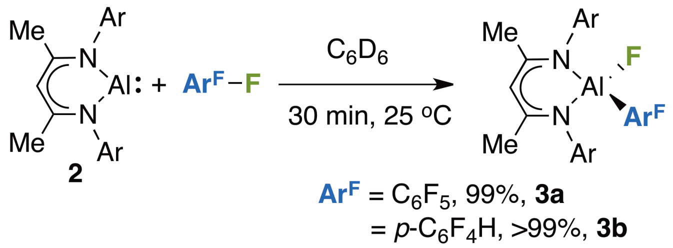

3a (R = C_6F_5) ^{1}H NMR (C_6D_6, 400 MHz, 298K) δ 7.10-7.08 (m, 4H), 6.89-6.86 (m, 2H), 4.99 (s, 1H), 3.40 (hept, 2H, $^3J_{H–H}$ = 6.8 Hz), 2.91 (hept, 2H, $^3J_{H–H}$ = 6.8 Hz), 1.48 (s, 6H), 1.47 (d, 6H, $^3J_{H–H}$ = 6.8 Hz), 1.09 (d, 6H, $^3J_{H–H}$ = 6.8 Hz), 0.99 (d, 6H, $^3J_{H–H}$ = 6.8 Hz), 0.45 (d, 6H, $^3J_{H–H}$ = 6.8 Hz); ^{19}F NMR (C_6D_6, 470.5 Hz, 298 K) δ -118.7 (m, 2F, *ortho-Ar*), -153.4 (t, 1F, $^3J_{F–F}$ = 23.5 Hz, *para-Ar*), -162.1 (m, 2F, *meta-Ar*), -168.2 (broad s, 1F, Al–F).^{13}C NMR (C_6D_6, 125 MHz, 298K) δ 171.9 (*C*=N), 151.3 (dm, $^1J_{F\text{-}C}$ = 265.0 Hz, Ar^F), 136.9 (dm, $^1J_{F\text{-}F}$ = 313.7 Hz, Ar^F), 144.9 (q Ar), 144.2 (q Ar), 137.9 (q Ar), 128.1 (Ar), 125.0 (Ar), 124.8 (Ar), 98.7 (*C*HC=N), 29.0 (*C*HMe$_2$), 27.8 (*C*HMe$_2$), 24.9 (CH*Me*$_2$), 24.5 (2 signals, CH*Me*$_2$), 24.2 (CH*Me*$_2$), 23.2 (*Me*), *remaining carbons of* C_6F_5 unit not resolved due to multiple coupling to ^{19}F (I=1/2) and line broadening by ^{27}Al (I=5/2). Data match those reported previously.[2]

3b (R = 4-C_6F_4H) ^{1}H NMR (C_6D_6, 400 MHz, 298K) δ 7.10-7.08 (m, 4H), 6.92-6.89 (m, 2H), 6.49-6.40 (m, 1H), 5.01 (s, 1H), 3.43 (hept, 2H, $^3J_{H–H}$ = 6.8 Hz), 3.01 (hept, 2H, $^3J_{H–H}$ = 6.8 Hz), 1.51 (s, 6H), 1.48 (d, 6H, $^3J_{H–H}$ = 6.8 Hz), 1.11 (d, 6H, $^3J_{H–H}$ = 6.8 Hz), 1.03 (d, 6H, $^3J_{H–H}$ = 6.8 Hz), 0.49 (d, 6H, $^3J_{H–H}$ = 6.8 Hz); ^{13}C NMR (C_6D_6, 125 MHz, 298K) δ 171.8 (*C*=N), 151.5 (dm, $^1J_{F\text{-}F}$ = 237.5 Hz, Ar^F), 146.2 (dm, $^1J_{F\text{-}F}$ = 250.0 Hz, Ar^F), 144.9 (q Ar), 144.4 (q Ar), 138.1 (q Ar), 128.1 (Ar), 124.9 (Ar), 124.7 (Ar), 107.3 (t, Ar^FH, $^2J_{F\text{-}F}$ = 22.9 Hz), 97.6 (*C*HC=N), 29.0 (*C*HMe$_2$), 27.8 (*C*HMe$_2$), 25.0 (CH*Me*$_2$), 24.6 (CH*Me*$_2$), 24.5 (CH*Me*$_2$), 24.3 (CH*Me*$_2$), 23.2 (*Me*), *ipso* carbon of C_6F_5H unit not resolved due to multiple coupling to ^{19}F (I=1/2) and line broadening by ^{27}Al (I=5/2);^{19}F NMR (C_6D_6, 470.5 Hz, 298 K) δ -120.5 (m, 2F, *ortho-Ar*), -139.8 (m, 2F, *meta-Ar*), -168.5 (broad s, 1F, Al–F). Mass spec (EI, +ve) 612 (40%, M^+), 463 (100%, $[M\text{-}C_6F_5H]^+$). HRMS calc. for $C_{35}H_{42}N_2F_5Al$ 612.3084, found 612.3076.

---

[2] S. Yow, S. J. Gates, A. J. P. White, M. R. Crimmin, *Angew. Chem., Int. Ed.* 2012, **51**, 12559.

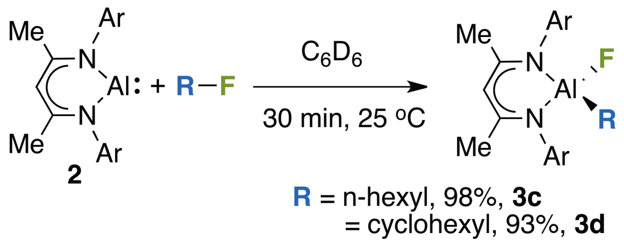

3c (R = n-hexyl): ^{1}H NMR (C_6D_6, 400 MHz, 298K) δ 7.15-7.13 (m, 4H), 7.08-7.06 (m, 2H), 4.97 (s, 1H), 3.62 (hept, 2H, $^3J_{H–H}$ = 6.8 Hz), 3.17 (hept, 2H, $^3J_{H–H}$ = 6.8 Hz), 1.57 (s, 6H), 1.47 (d, 6H, $^3J_{H–H}$ = 6.8 Hz), 1.35 (d, 6H, $^3J_{H–H}$ = 6.8 Hz),1.17 (d, 6H, $^3J_{H–H}$ = 6.8 Hz), 1.10-1.01 (m, 8H), 1.05 (d, 6H, $^3J_{H–H}$ = 6.8 Hz), 0.76 (t, 3H, $^3J_{H–H}$ = 7.2 Hz), -0.14 (m, 2H); ^{13}C NMR (C_6D_6, 125 MHz, 298K) δ δ 170.2 (*C*=N), 145.5 (q Ar),143.3 (q Ar), 140.2 (q Ar), 127.6 (Ar), 125.1 (Ar), 124.0 (Ar), 97.7 (*C*HC=N), 35.4 (*hex*CH$_2$), 31.9 (*hex*CH$_2$), 29.0 (*C*HMe$_2$), 27.9 (*C*HMe$_2$), 25.8 (CH*Me*$_2$), 25.1 (*hex*CH$_2$), 24.8 (CH*Me*$_2$), 24.4 (CH*Me*$_2$), 24.0 (CH*Me*$_2$), 23.0 (*Me*), 22.8 (*hex*CH$_2$), 14.3 ((*hex*CH$_3$), 5.9 (broad, Al–*C*);^{19}F NMR (C_6D_6, 470.5 Hz, 298 K) δ -157.7 (broad s, 1F, Al–F). Mass spec (EI, +ve) 527 (100%, $[M\text{-}F]^+$), 463 (95%, $[M\text{-}C_6H_{11}]^+$). HRMS calc. for $C_{35}H_{52}N_2Al$ 527.3946 found 527.3959.

3d (R = Cy): ^{1}H NMR (C_6D_6, 400 MHz, 298K) δ 7.15-7.07 (m, 6H), 4.96 (s, 1H), 3.58 (hept, 2H, $^3J_{H–H}$ = 6.8 Hz), 3.24 (hept, 2H, $^3J_{H–H}$ = 6.8 Hz), 1.56 (s, 6H), 1.55-1.48 (m, 2H), 1.48 (d, 6H, $^3J_{H–H}$ = 6.8 Hz), 1.39 (d, 6H, $^3J_{H–H}$ = 6.8 Hz), 1.32-1.16 (m, 4H), 1.15 (d, 6H, $^3J_{H–H}$ = 6.8 Hz), 1.08-1.01 (m, 2H), 1.07 (d, 6H, $^3J_{H–H}$ = 6.8 Hz), 0.76-0.73 (broad m, 2H), 0.29-0.20 (broad m, 1H); ^{13}C NMR (C_6D_6, 125 MHz, 298K) δ 170.2 (*C*=N), 145.4 (q Ar),143.2 (q Ar), 140.6 (q Ar), 127.6 (Ar), 125.2 (Ar), 123.8 (Ar), 97.6 (*C*HC=N), 30.0 (*cy*CH$_2$), 29.4 (*cy*CH$_2$), 29.0 (*C*HMe$_2$), 28.0 (*cy*CH$_2$), 27.9 (*C*HMe$_2$), 25.5 (CH*Me*$_2$), 24.9 (CH*Me*$_2$), 24.5 (CH*Me*$_2$), 23.9 (CH*Me*$_2$), 23.0 (*Me*), 21.2 (broad, Al–*C*); ^{19}F NMR (C_6D_6, 470.5 Hz, 298 K) δ -161.8 (broad s, 1F, Al–F). Mass spec (EI, +ve) 548 (100%, $[M]^+$), 463 (60%, $[M\text{-}C_6H_{13}]^+$). HRMS calc. for $C_{35}H_{54}N_2FAl$ 548.40846 found 548.4077.

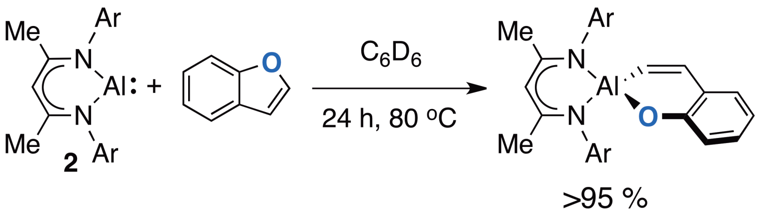

4 ^{1}H NMR (C_6D_6, 500 MHz, 298K) δ 7.43 (d, 1H, $^{3}J_{H–H}$ = 16.0 Hz), 7.12 (dd, 1H, $^{3}J_{H–H}$ = 8.0, 1.5 Hz), 7.07 (ddd, 1H, $^{3}J_{H–H}$ = 8.0, 7.0, 1.5), 7.01-6.99 (m, 4H), 6.94-6.92 (m, 2H), 6.83 (dd, 1H, $^{3}J_{H–H}$ = 7.0, 1.5 Hz) 6.67 (ddd, 1H, $^{3}J_{H–H}$ = 8.0, 7.0,1.5 Hz), 5.98 (d, 1H, $^{3}J_{H–H}$ = 16.0 Hz) 4.97 (s, 1H), 3.63 (hept, 2H, $^{3}J_{H–H}$ = 6.8 Hz), 3.28 (hept, 2H, $^{3}J_{H–H}$ = 6.8 Hz), 1.54 (s, 6H), 1.38 (d, 6H, $^{3}J_{H–H}$ = 6.8 Hz), 1.15 (d, 6H, $^{3}J_{H–H}$ = 6.8 Hz), 1.09 (d, 6H, $^{3}J_{H–H}$ = 6.8 Hz), 1.03 (d, 6H, $^{3}J_{H–H}$ = 6.8 Hz), ^{13}C NMR (C_6D_6, 125 MHz, 298K) δ 170.7 (C=N), 160.1 (q, ArCH=CH), 153.6 (ArCH=CH),144.5 (q Ar), 143.2 (q Ar), 139.6 (q Ar), 131-132 (broad, ArCH=CH), 132.1 (ArCH=CH), 128.3 (Ar), 127.7 (ArCH=CH), 127.6 (ArCH=CH),125.0 (Ar), 124.0 (Ar), 120.8 (ArCH=CH), 118.0 (ArCH=CH), 98.3 (CHC=N), 29.1 (CHMe2), 28.4 (CHMe2), 26.1 (CHMe2), 24.8 (CHMe2), 24.7 (CHMe2), 24.4 (CHMe2), 23.1 (Me).

X-ray Crystallography Data

Crystal data for **3a**: $C_{35}H_{41}AlF_6N_2$, M = 630.68, monoclinic, $P2_1/c$ (no. 14), a = 22.5345(3), b = 17.93193(19), c = 26.6666(4) Å, β = 110.2781(15)°, V = 10107.8(2) Å^3, Z = 12 [3 independent molecules], D_c = 1.243 g cm^{-3}, μ(Cu-Kα) = 1.036 mm^{-1}, T = 173 K, colourless blocks, Agilent Xcalibur PX Ultra A diffractometer; 19403 independent measured reflections (R_{int} = 0.0289), F^2 refinement,[X1] R_1(obs) = 0.0419, wR_2(all) = 0.1156, 14330 independent observed absorption-corrected reflections [$|F_o| > 4\sigma(|F_o|)$, $2\theta_{max}$ = 148°], 1219 parameters. CCDC 1419650.

Crystal data for **4**: $C_{37}H_{47}AlN_2O$, M = 562.74, orthorhombic, $Pna2_1$ (no. 33), a = 16.8019(4), b = 12.4929(3), c = 15.8089(4) Å, V = 3318.34(14) Å^3, Z = 4, D_c = 1.126 g cm^{-3}, μ(Mo-Kα) = 0.091 mm^{-1}, T = 173 K, colourless blocks, Agilent Xcalibur 3E diffractometer; 4227 independent measured reflections (R_{int} = 0.0264), F^2 refinement,[3] R_1(obs) = 0.0365, wR_2(all) = 0.0865, 3752 independent observed absorption-corrected reflections [$|F_o| > 4\sigma(|F_o|)$, $2\theta_{max}$ = 57°], 380 parameters. The absolute structure of **4** was determined by use of the Flack parameter [x = 0.14(12)]. CCDC 1419651.

The structure of **3a** was found to contain three crystallographically independent complexes, **3a-A**, **3a-B** and **3a-C**. The absolute structure of **4** was determined by use of the Flack parameter [x = 0.14(12)].

[3] (a) SHELXTL, Bruker AXS, Madison, WI; (b) SHELX-2013, http://shelx.uni-ac.gwdg.de/SHELX/index.php

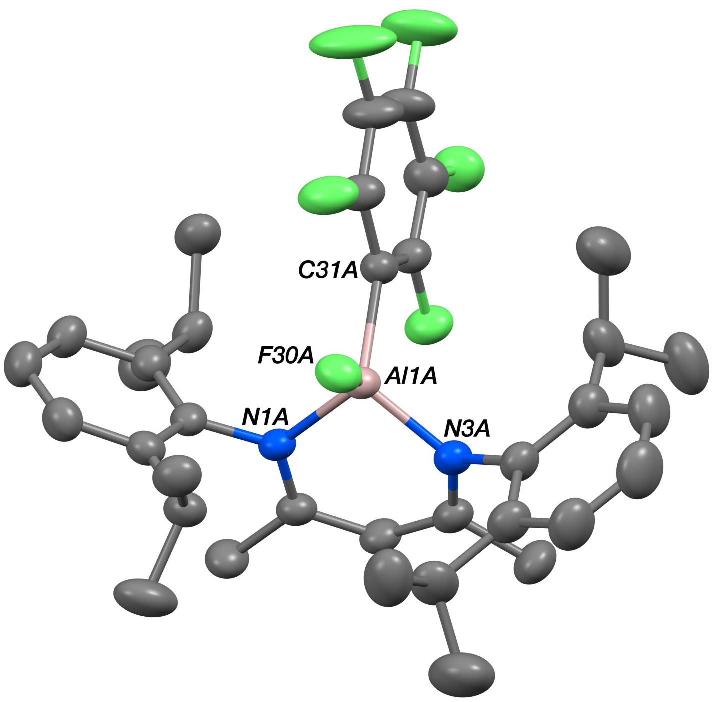

Fig. S1 The structure of one (**3a-A**) of the three independent complexes present in the crystal of **3a** (50% probability ellipsoids).

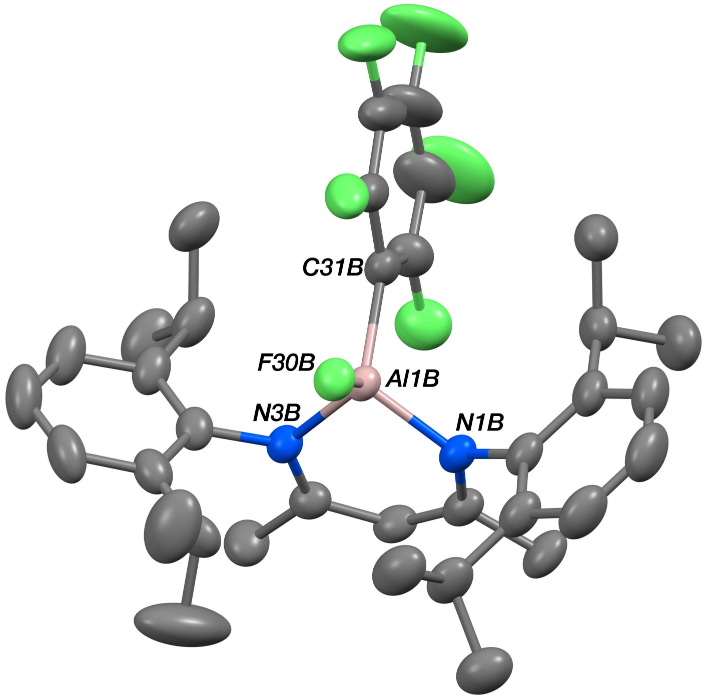

Fig. S2 The structure of one (**3a-B**) of the three independent complexes present in the crystal of **3a** (50% probability ellipsoids).

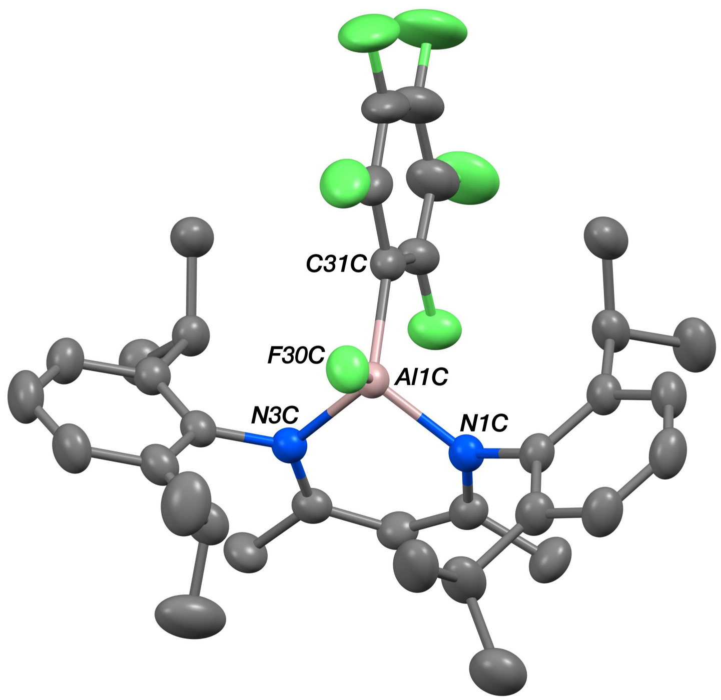

Fig. S3 The structure of one (**3a-C**) of the three independent complexes present in the crystal of **3a** (50% probability ellipsoids).

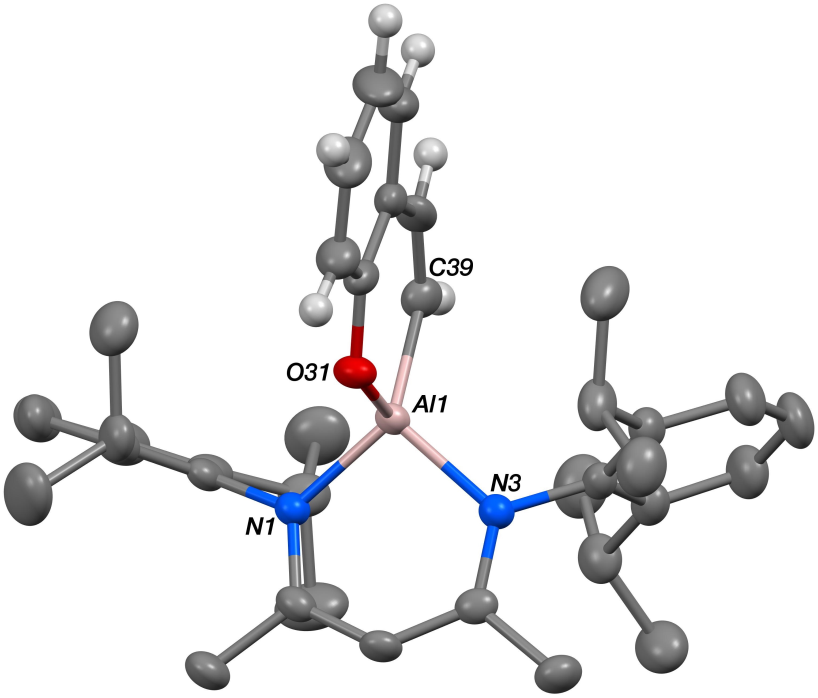

Fig. S4 The crystal structure of **4** (50% probability ellipsoids).

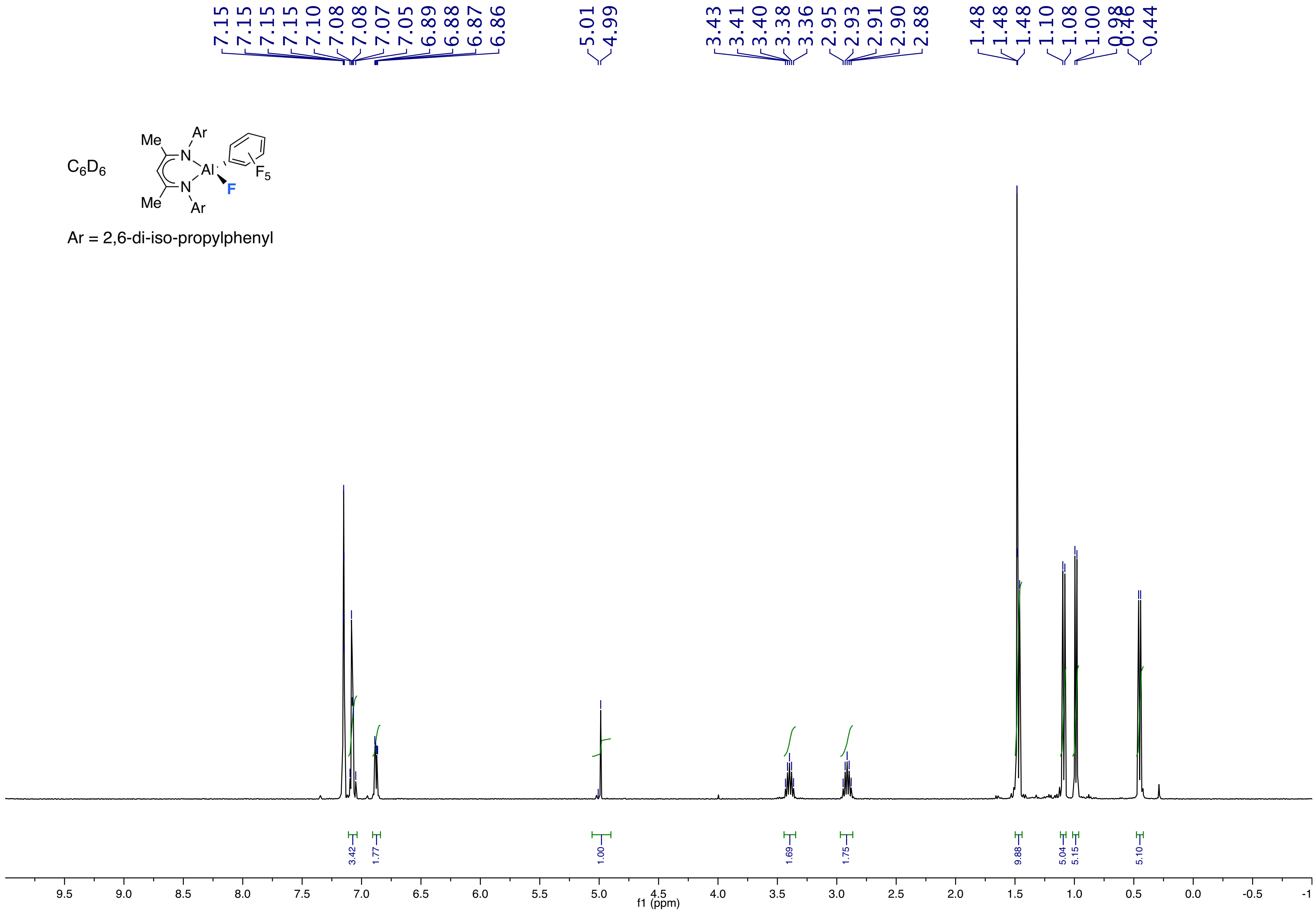
7.15 7.15 7.15 7.15 7.10 7.08 7.08 7.07 7.05 6.89 6.88 6.87 6.86
5.01 4.99
3.43 3.41 3.40 3.38 3.36 2.95 2.93 2.91 2.90 2.88
1.48 1.48 1.48 1.10 1.08 1.00 0.98 0.46 0.44
C6D6
Me
Ar
N
Al
F5
F
Me
Ar
Ar = 2,6-di-iso-propylphenyl
3.42
1.77
1.00
1.69
1.75
9.88
5.04
5.15
5.10
9.5 9.0 8.5 8.0 7.5 7.0 6.5 6.0 5.5 5.0 4.5 4.0 3.5 3.0 2.5 2.0 1.5 1.0 0.5 0.0 -0.5 -1
f1 (ppm)

C_6D_6

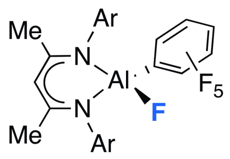

Ar = 2,6-di-iso-propylphenyl

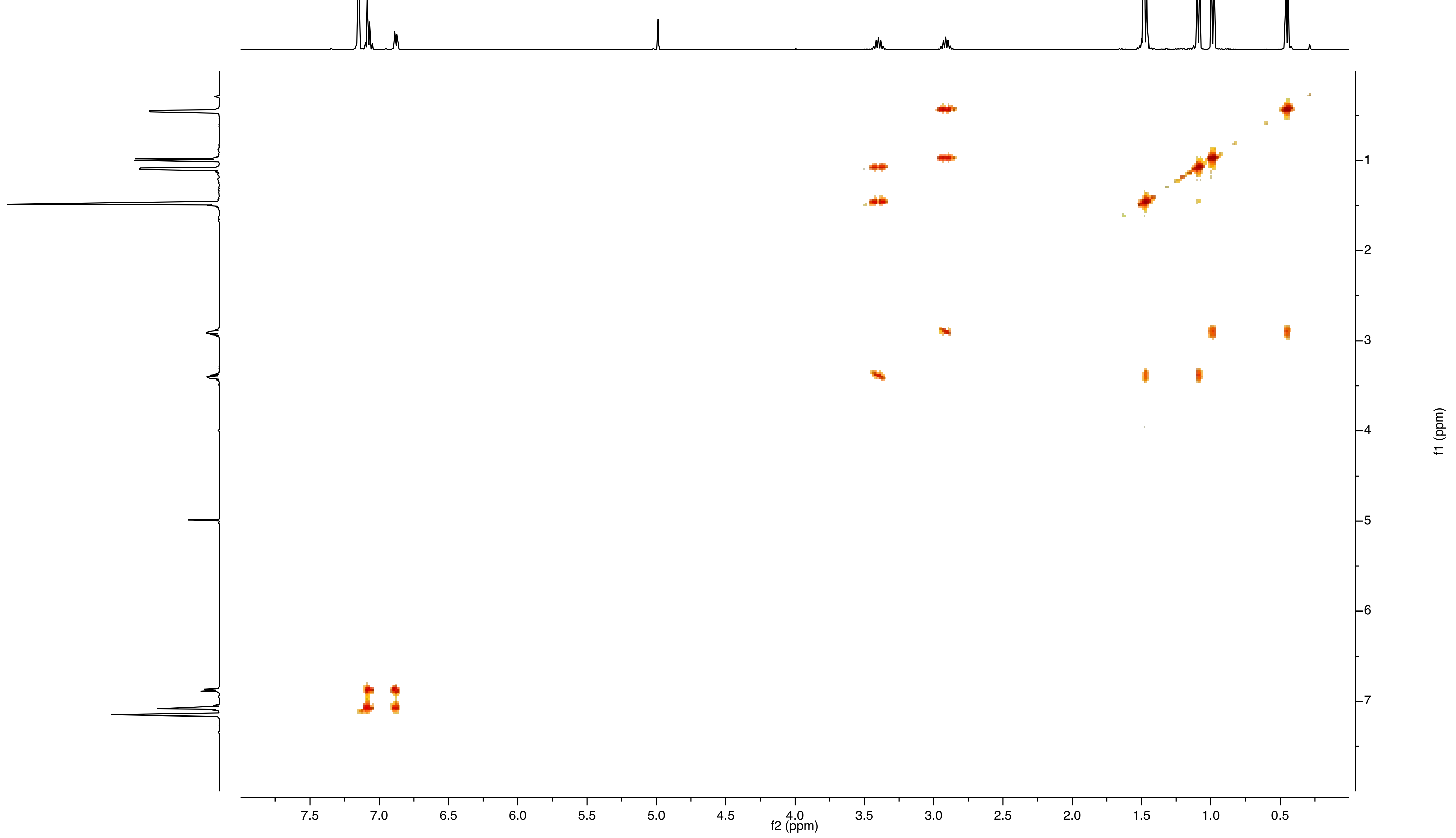

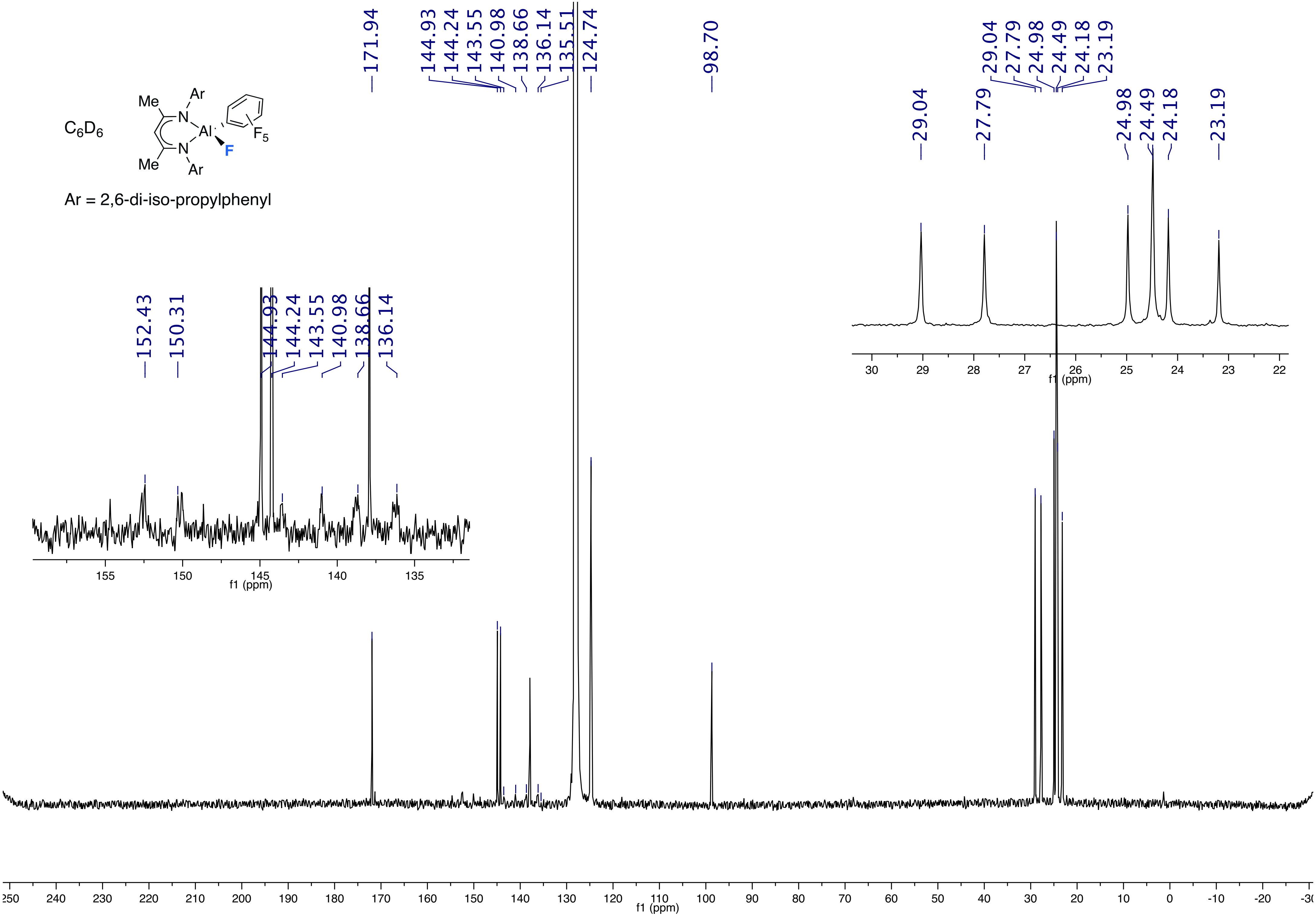
C6D6
Me
Ar
N
Al
F5
F
N
Me
Ar
Ar = 2,6-di-iso-propylphenyl
171.94
144.93
144.24
143.55
140.98
138.66
136.14
135.51
124.74
98.70
29.04
27.79
24.98
24.49
24.18
23.19
152.43
150.31
f1 (ppm)

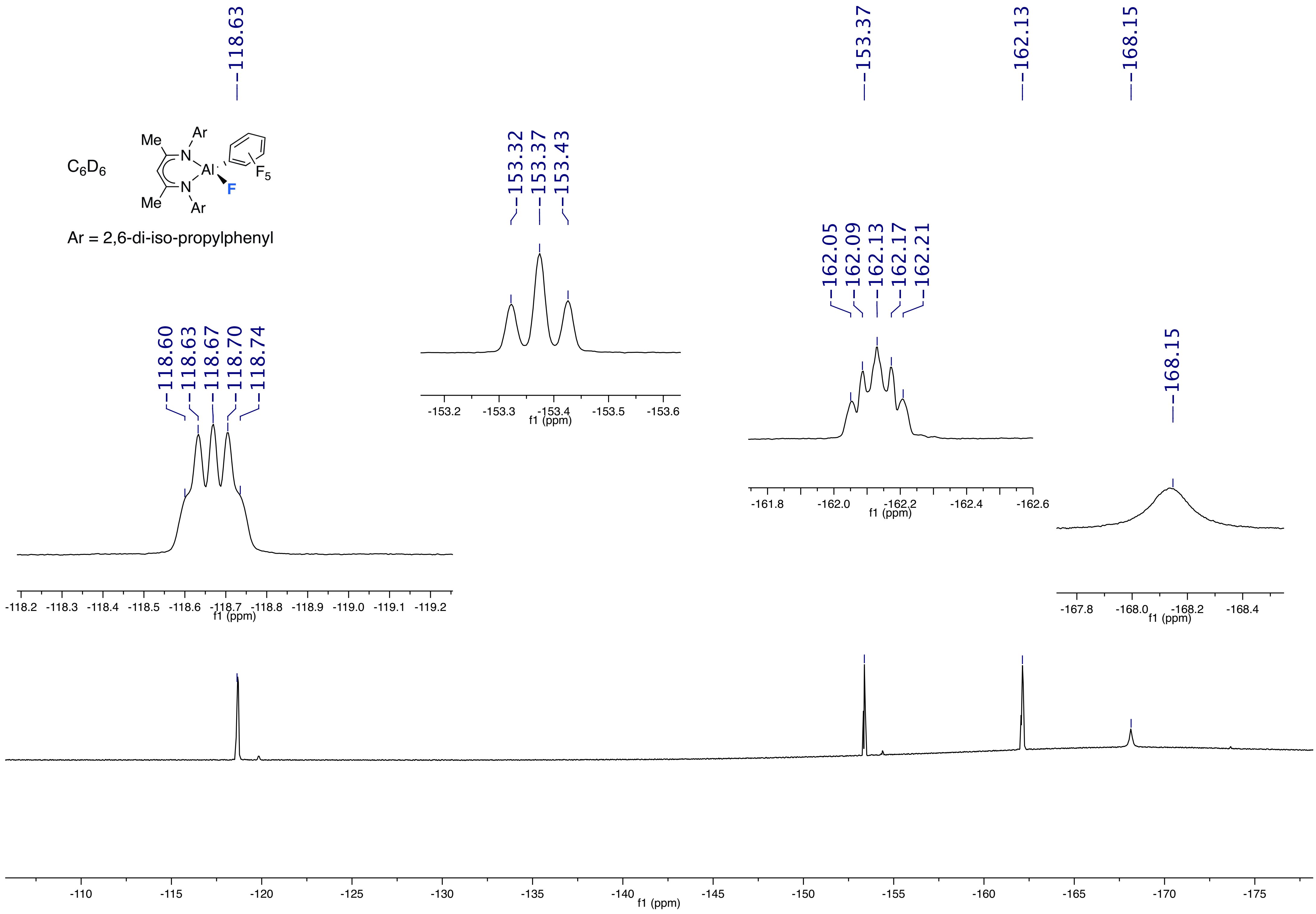

C6D6
Me
Ar
N
Al
F5
F
N
Me
Ar
Ar = 2,6-di-iso-propylphenyl
-118.63
-153.37
-162.13
-168.15
-118.60
-118.63
-118.67
-118.70
-118.74
-153.32
-153.37
-153.43
-162.05
-162.09
-162.13
-162.17
-162.21
-168.15
f1 (ppm)

C6D6

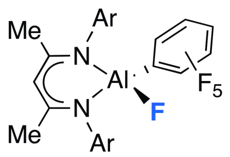

Ar = 2,6-di-iso-propylphenyl

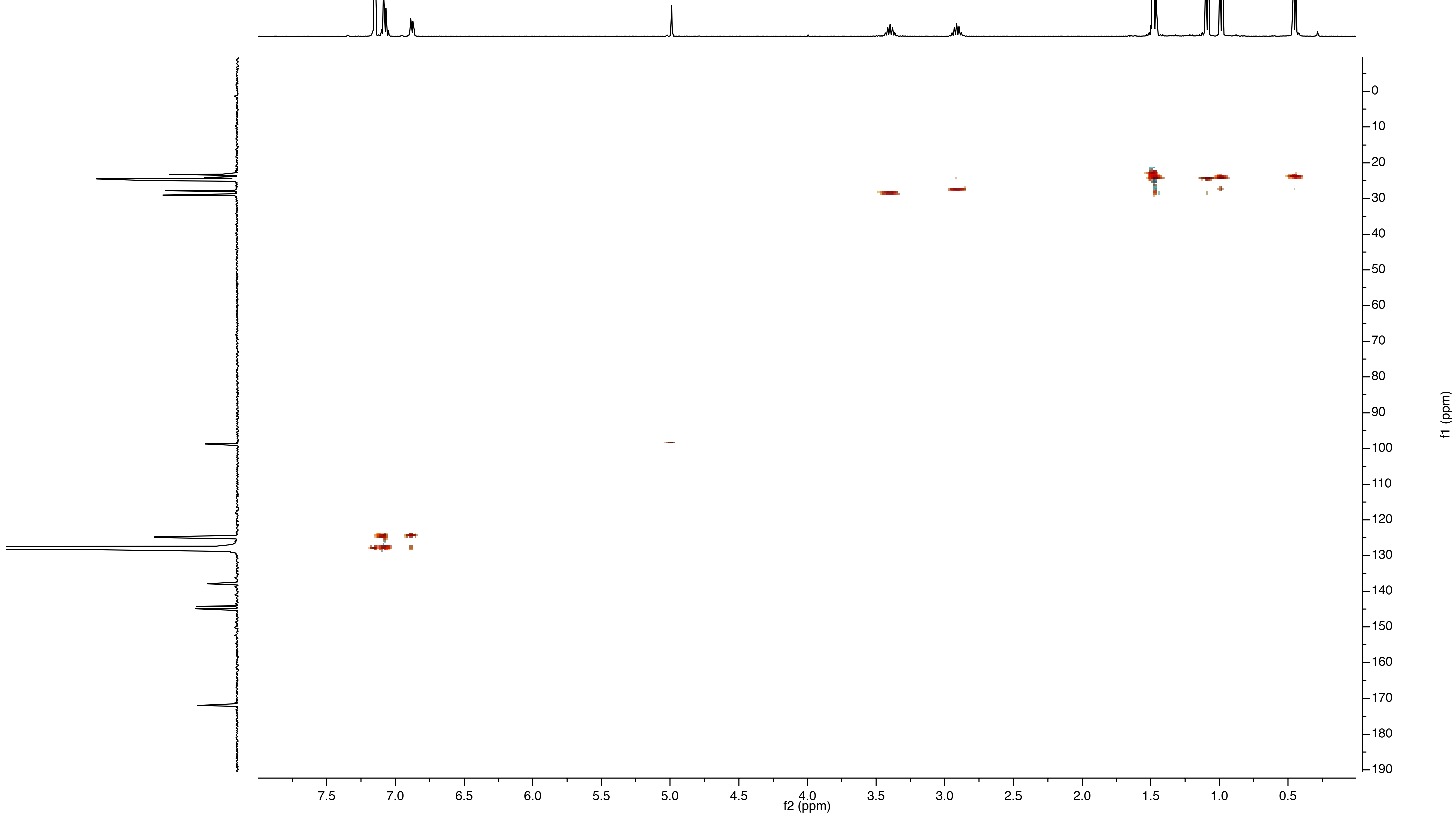

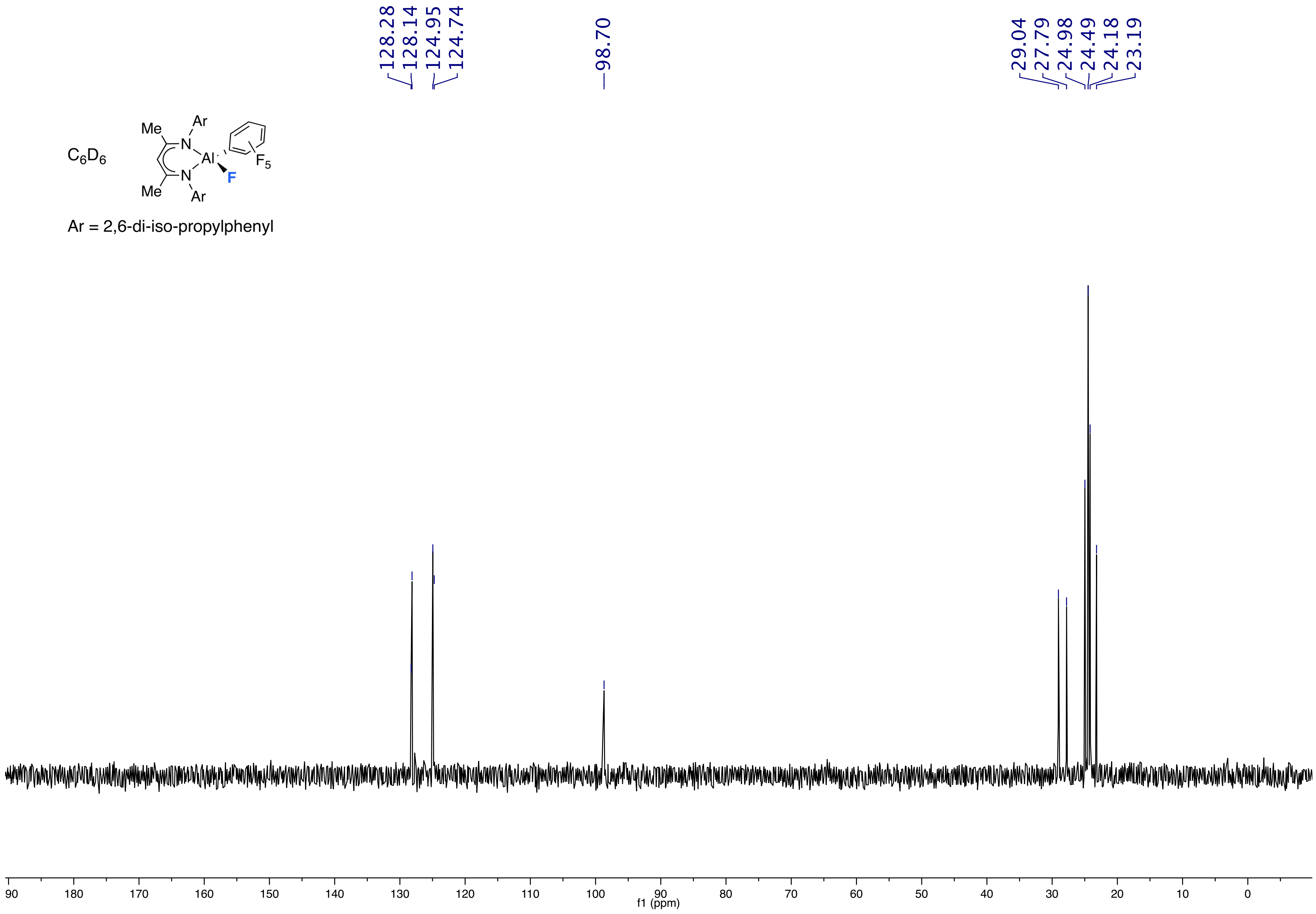
128.28
128.14
124.95
124.74
98.70
29.04
27.79
24.98
24.49
24.18
23.19
C_6D_6
Me
Ar
N
Al
F
F_5
Me
Ar
Ar = 2,6-di-iso-propylphenyl
90
180
170
160
150
140
130
120
110
100
90
80
70
60
50
40
30
20
10
0
f1 (ppm)

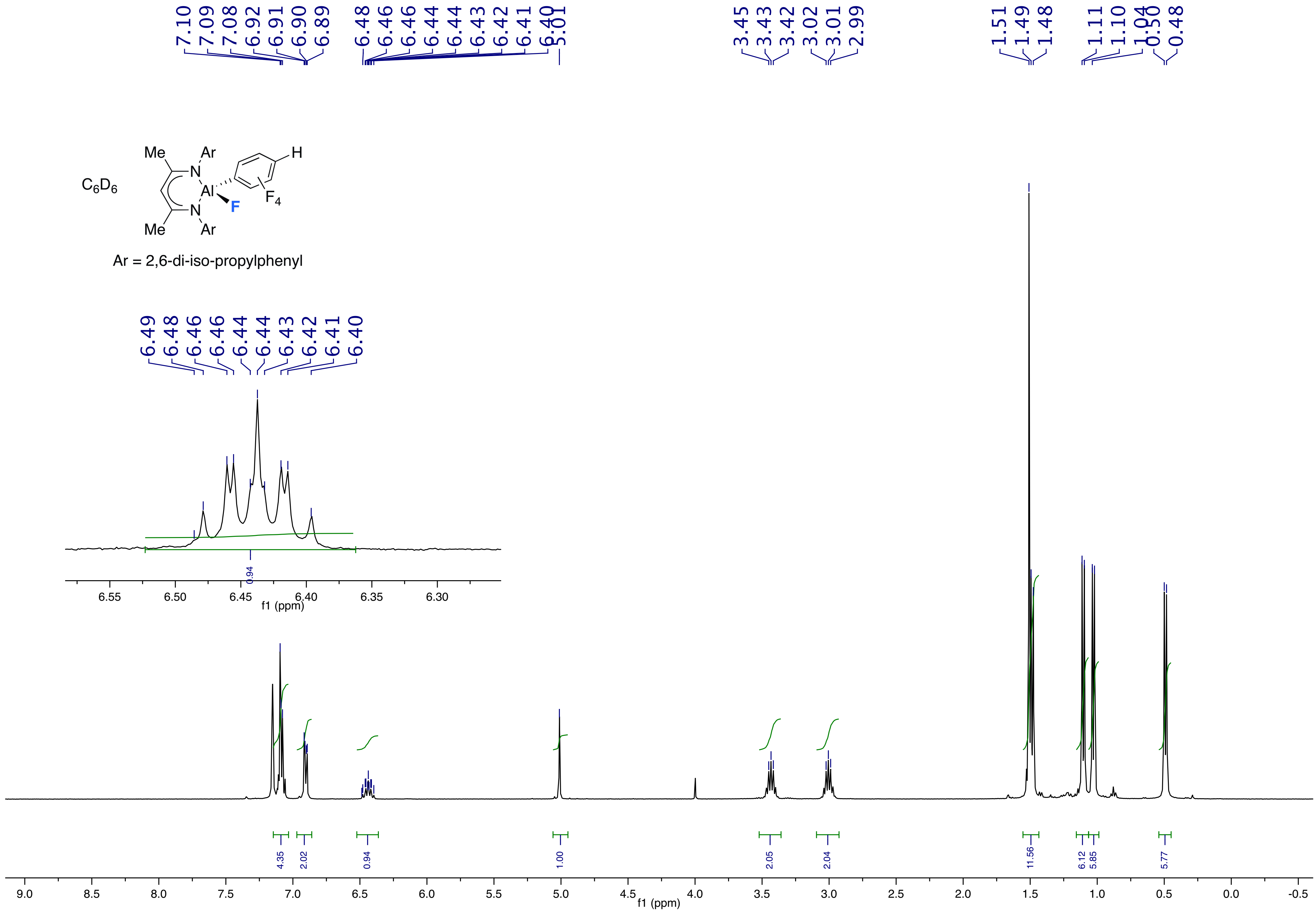

7.10 7.09 7.08 6.92 6.91 6.90 6.89 6.48 6.46 6.46 6.44 6.44 6.43 6.42 6.41 6.40 5.01 3.45 3.43 3.42 3.02 3.01 2.99 1.51 1.49 1.48 1.11 1.10 1.04 0.50 0.48
C6D6
Me
Ar
N
Al
F
H
F4
Me
Ar
Ar = 2,6-di-iso-propylphenyl
6.49 6.48 6.46 6.46 6.44 6.44 6.43 6.42 6.41 6.40
0.94
6.55 6.50 6.45 6.40 6.35 6.30
f1 (ppm)
4.35 2.02 0.94 1.00 2.05 2.04 11.56 6.12 5.85 5.77
9.0 8.5 8.0 7.5 7.0 6.5 6.0 5.5 5.0 4.5 4.0 3.5 3.0 2.5 2.0 1.5 1.0 0.5 0.0 -0.5
f1 (ppm)

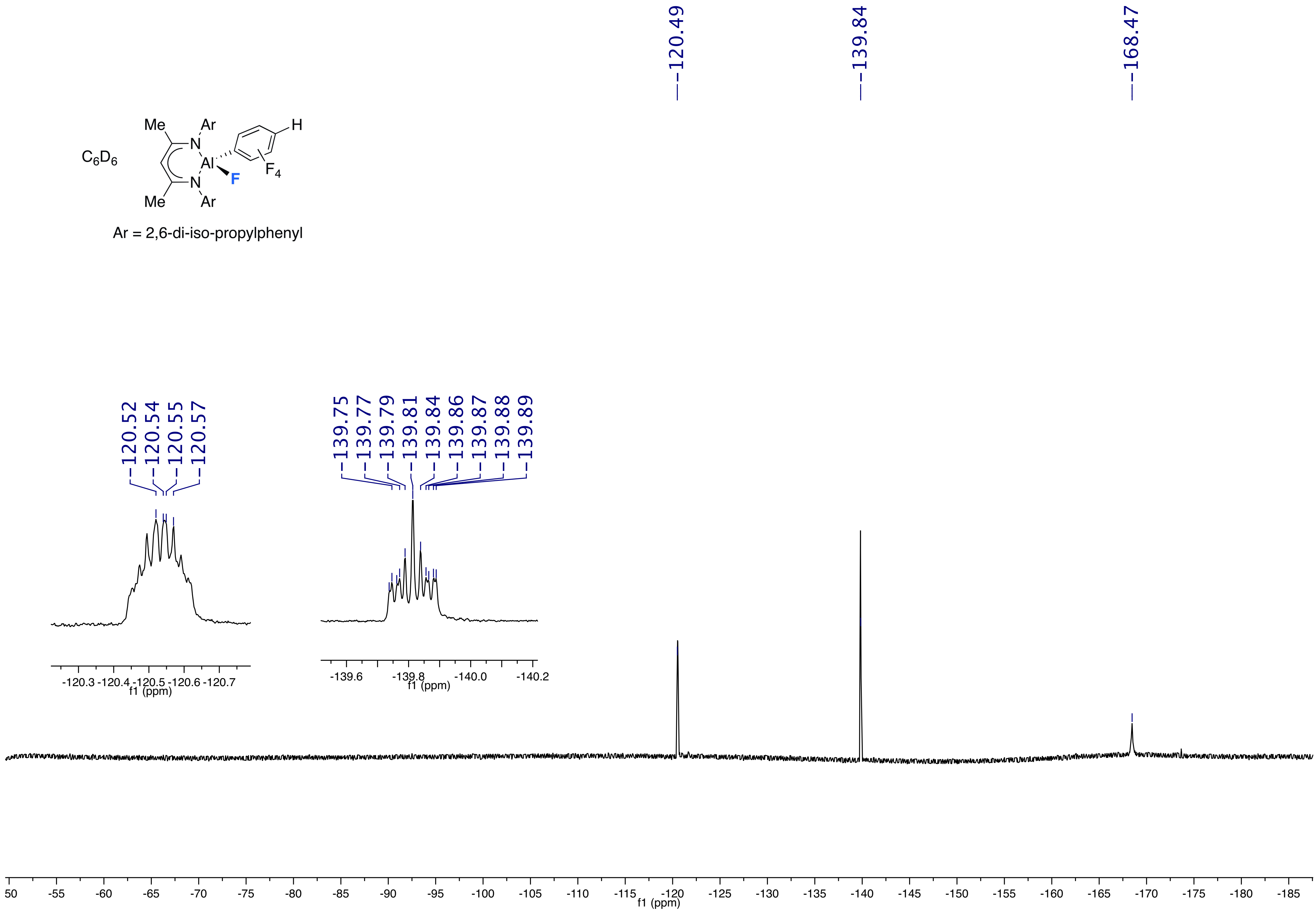

-120.49
-139.84
-168.47
C6D6
Me
Ar
N
Al
F
F4
H
Ar = 2,6-di-iso-propylphenyl
-120.52
-120.54
-120.55
-120.57
-139.75
-139.77
-139.79
-139.81
-139.84
-139.86
-139.87
-139.88
-139.89
-120.3 -120.4 -120.5 -120.6 -120.7
f1 (ppm)
-139.6 -139.8 -140.0 -140.2
f1 (ppm)
50 -55 -60 -65 -70 -75 -80 -85 -90 -95 -100 -105 -110 -115 -120 -125 -130 -135 -140 -145 -150 -155 -160 -165 -170 -175 -180 -185
f1 (ppm)

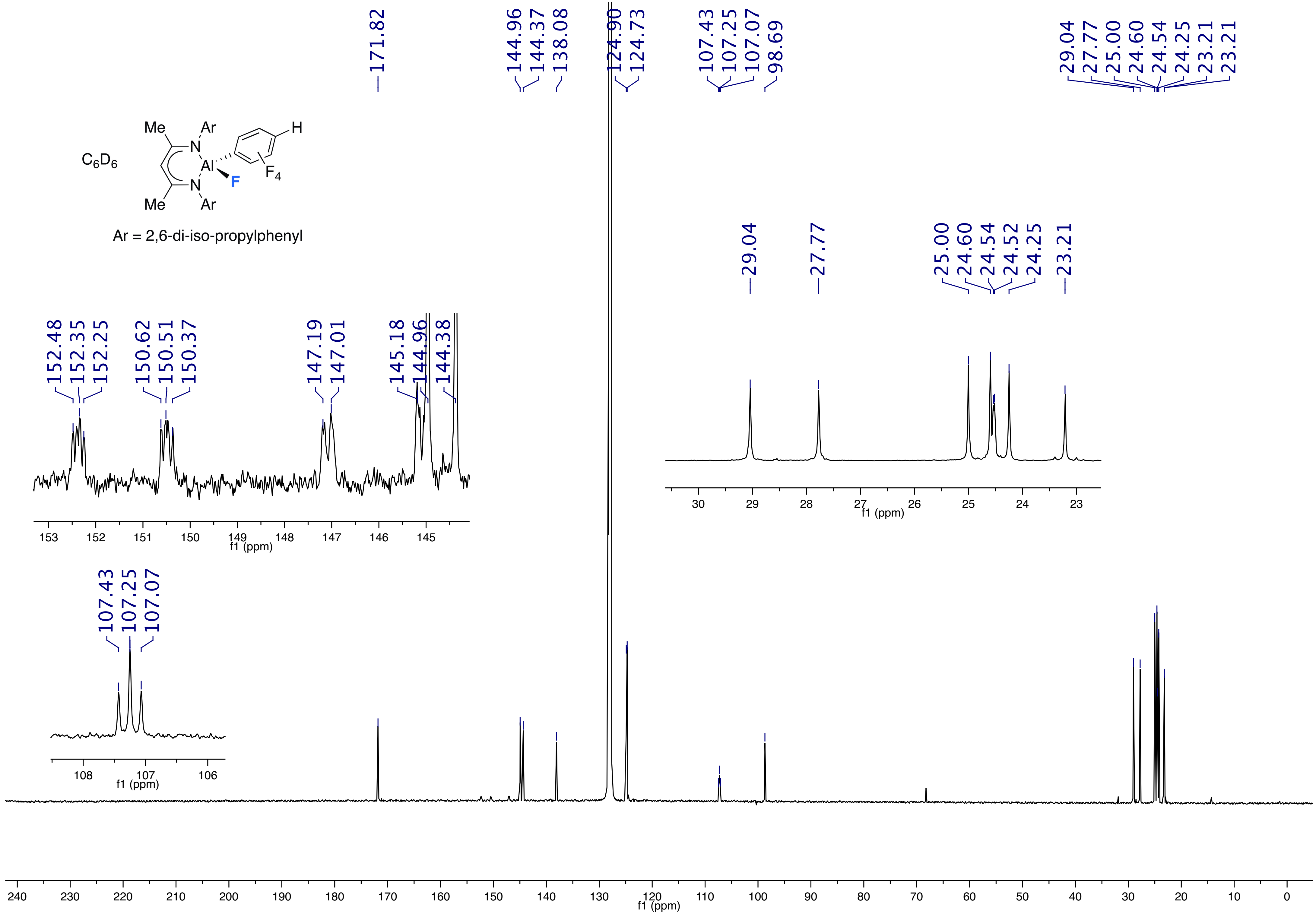

171.82
144.96
144.37
138.08
124.90
124.73
107.43
107.25
107.07
98.69
29.04
27.77
25.00
24.60
24.54
24.25
23.21
23.21
C_6D_6
Me
Ar
N
Al
F
H
F_4
Ar = 2,6-di-iso-propylphenyl
152.48
152.35
152.25
150.62
150.51
150.37
147.19
147.01
145.18
144.96
144.38
29.04
27.77
25.00
24.60
24.54
24.52
24.25
23.21
107.43
107.25
107.07
f1 (ppm)

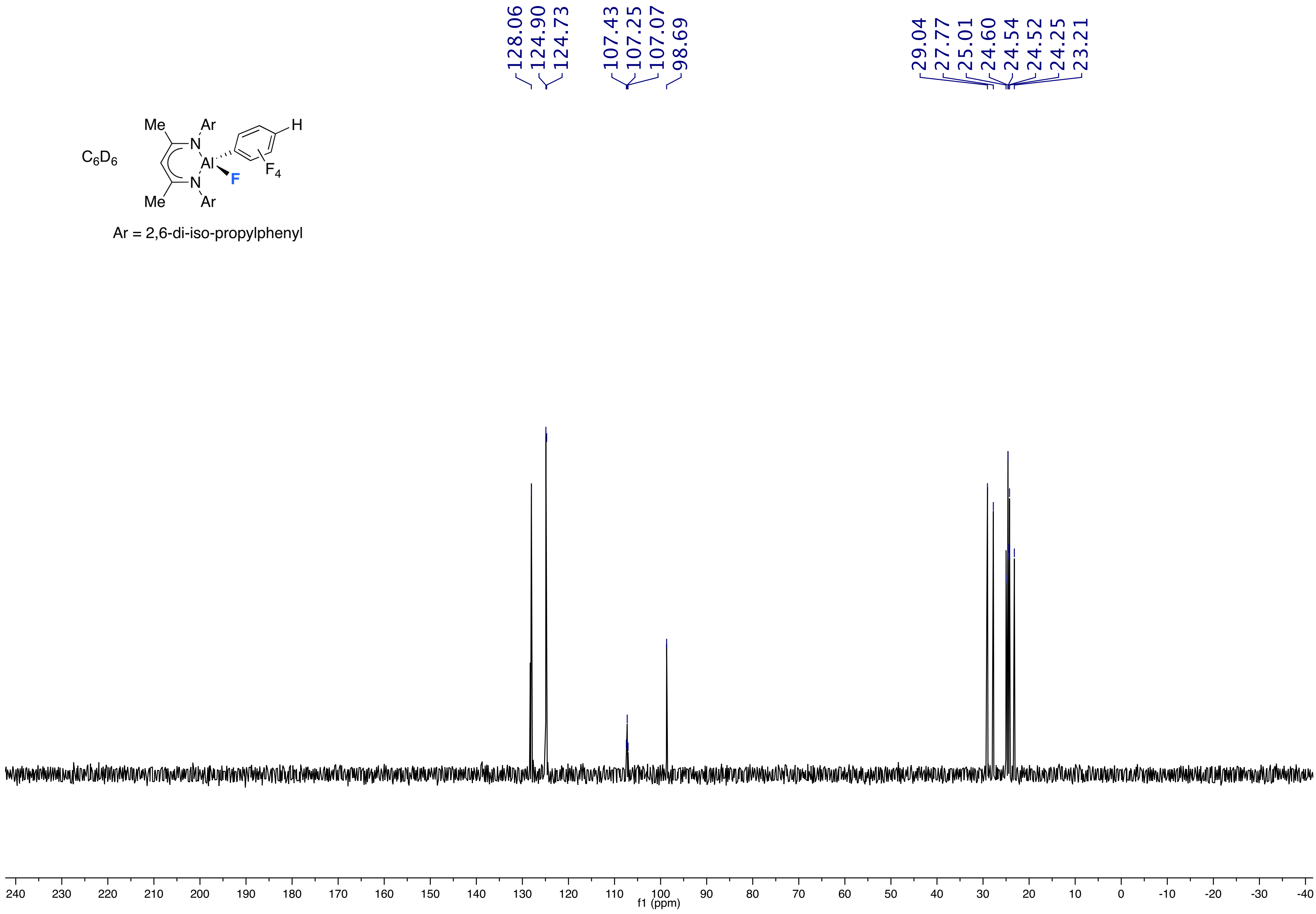
128.06
124.90
124.73
107.43
107.25
107.07
98.69
29.04
27.77
25.01
24.60
24.54
24.52
24.25
23.21
C6D6
Me
Ar
N
Al
F
F4
H
Me
Ar
N
Ar = 2,6-di-iso-propylphenyl
240 230 220 210 200 190 180 170 160 150 140 130 120 110 100 90 80 70 60 50 40 30 20 10 0 -10 -20 -30 -40
f1 (ppm)

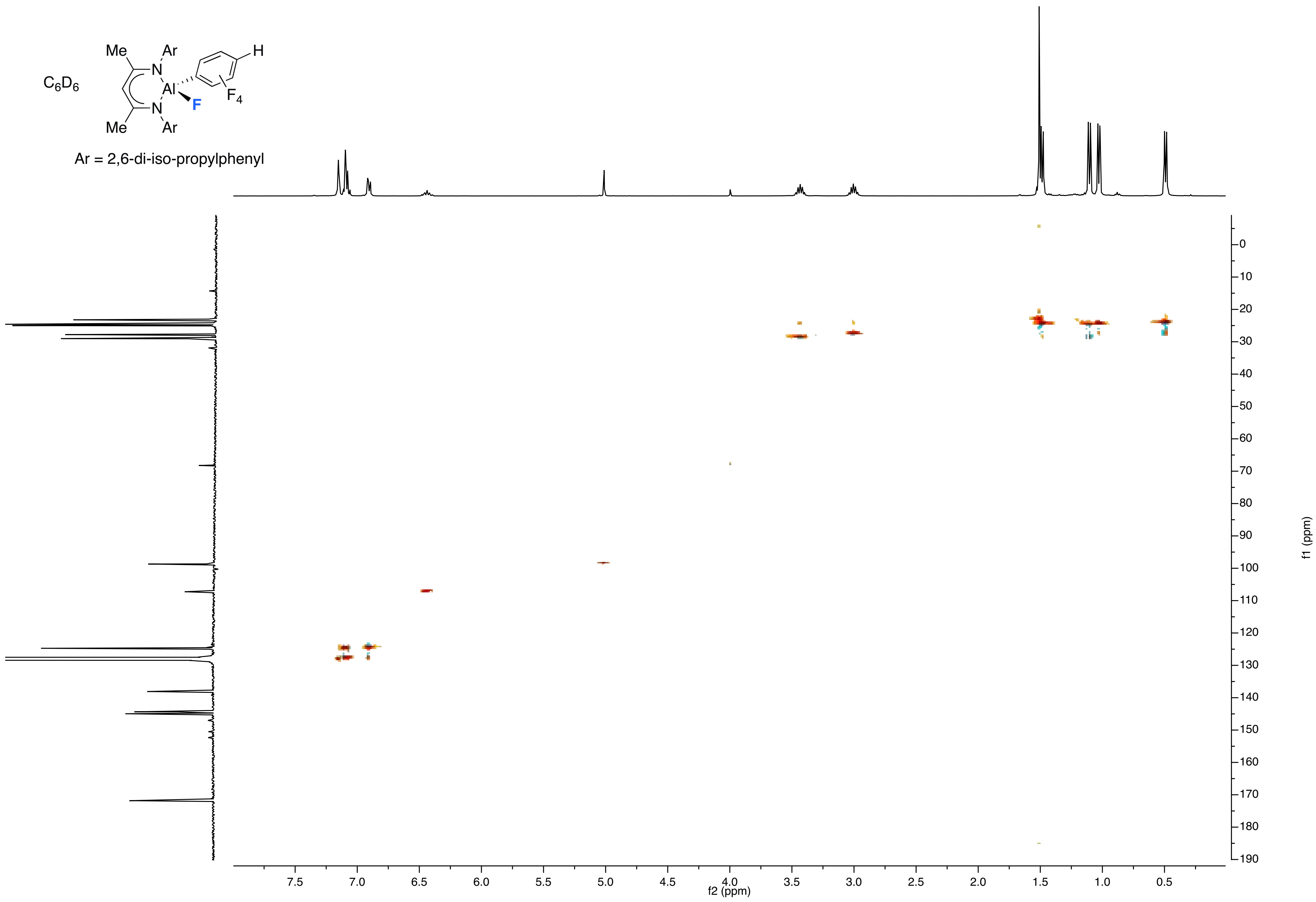

C6D6
Me
Ar
N
Al
F
H
F4
Me
Ar
Ar = 2,6-di-iso-propylphenyl
f2 (ppm)
f1 (ppm)

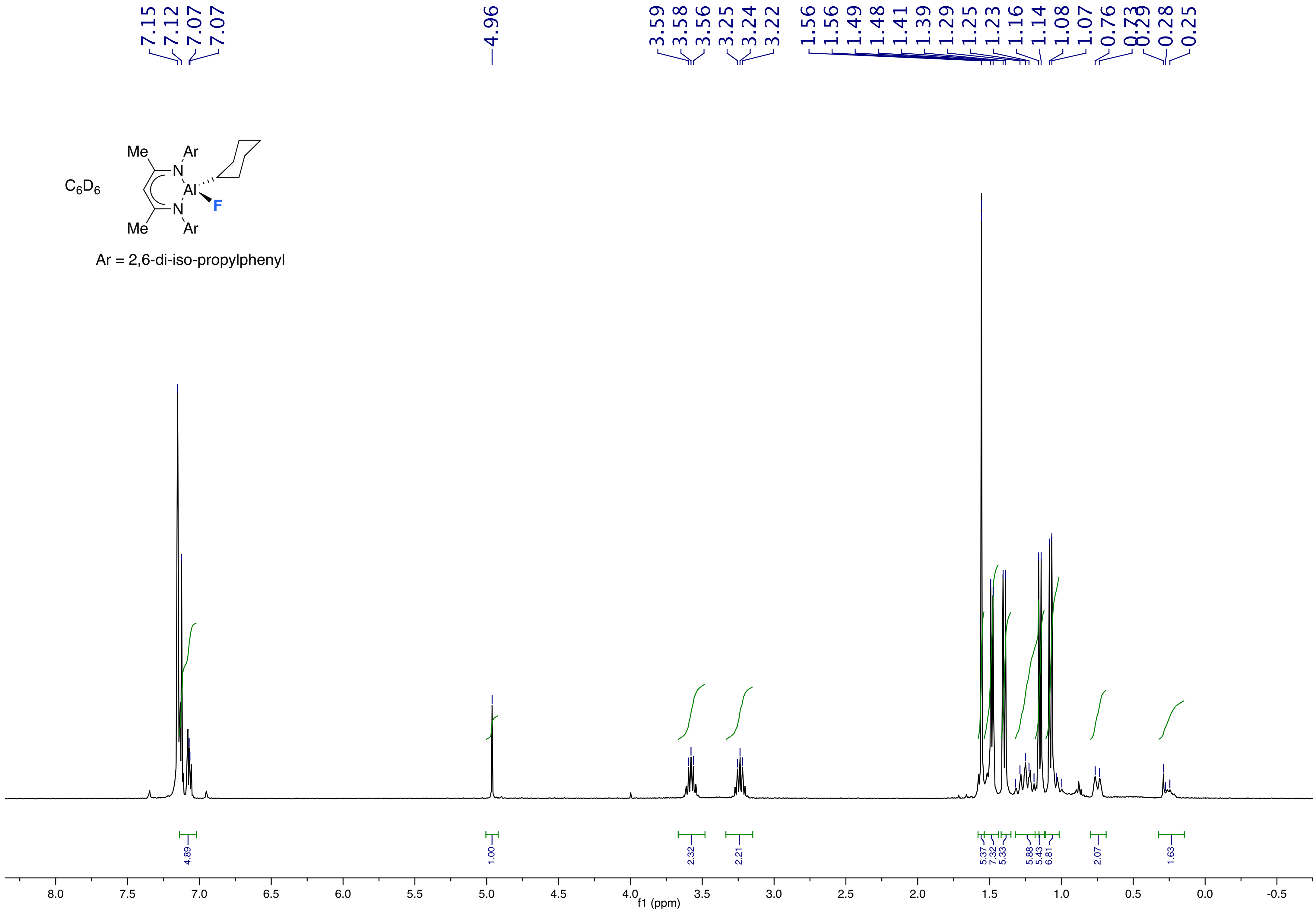

7.15
7.12
7.07
7.07
4.96
3.59
3.58
3.56
3.25
3.24
3.22
1.56
1.56
1.49
1.48
1.41
1.39
1.29
1.25
1.23
1.16
1.14
1.08
1.07
0.76
0.73
0.29
0.28
0.25
C6D6
Me
Ar
N
Al
F
N
Me
Ar
Ar = 2,6-di-iso-propylphenyl
4.89
1.00
2.32
2.21
5.37
7.32
5.33
5.88
5.43
6.81
2.07
1.63
8.0
7.5
7.0
6.5
6.0
5.5
5.0
4.5
4.0
3.5
3.0
2.5
2.0
1.5
1.0
0.5
0.0
-0.5
f1 (ppm)

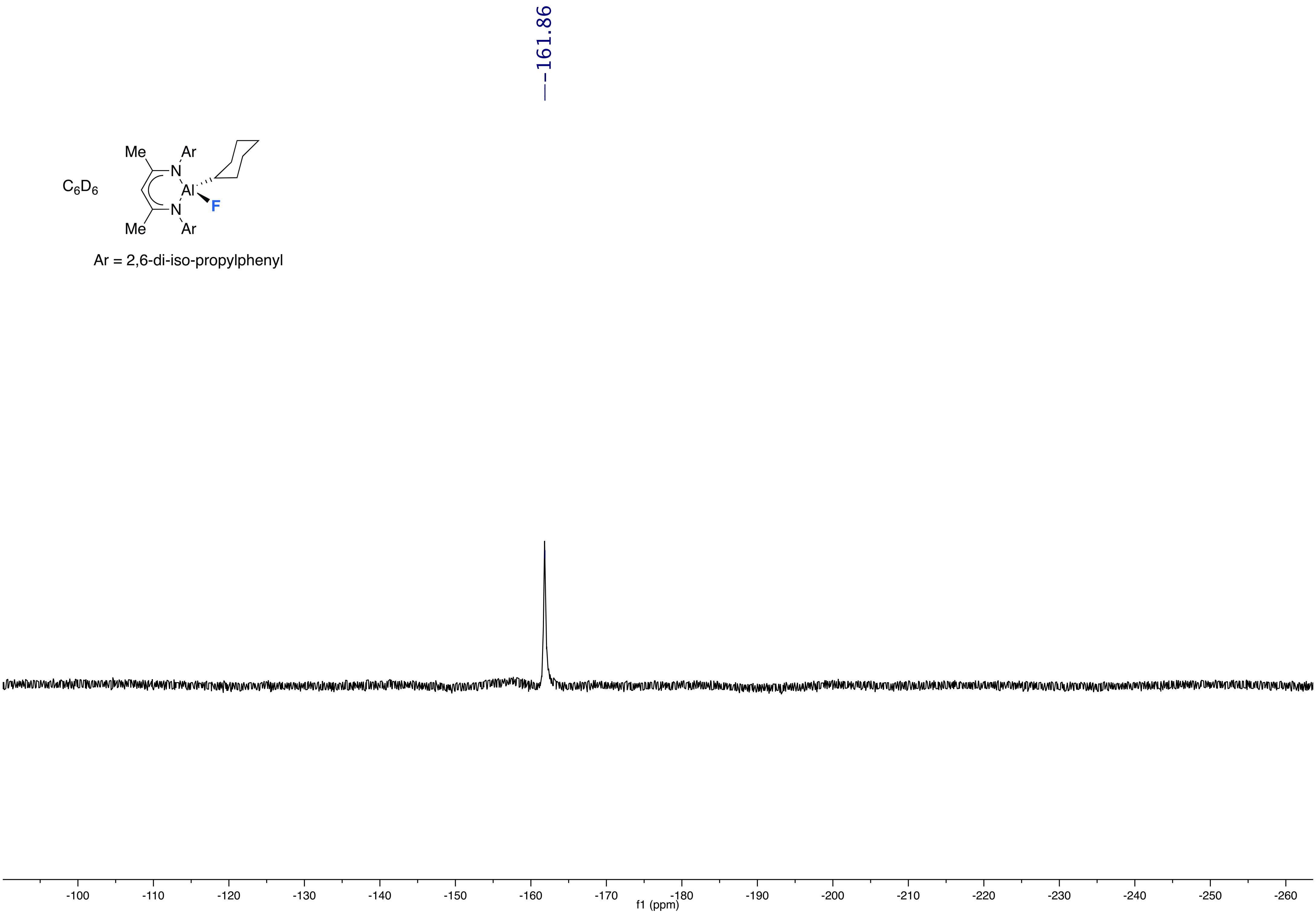

-161.86
C6D6
Me
Ar
N
Al
F
N
Me
Ar
Ar = 2,6-di-iso-propylphenyl
-100
-110
-120
-130
-140
-150
-160
-170
-180
-190
-200
-210
-220
-230
-240
-250
-260
f1 (ppm)

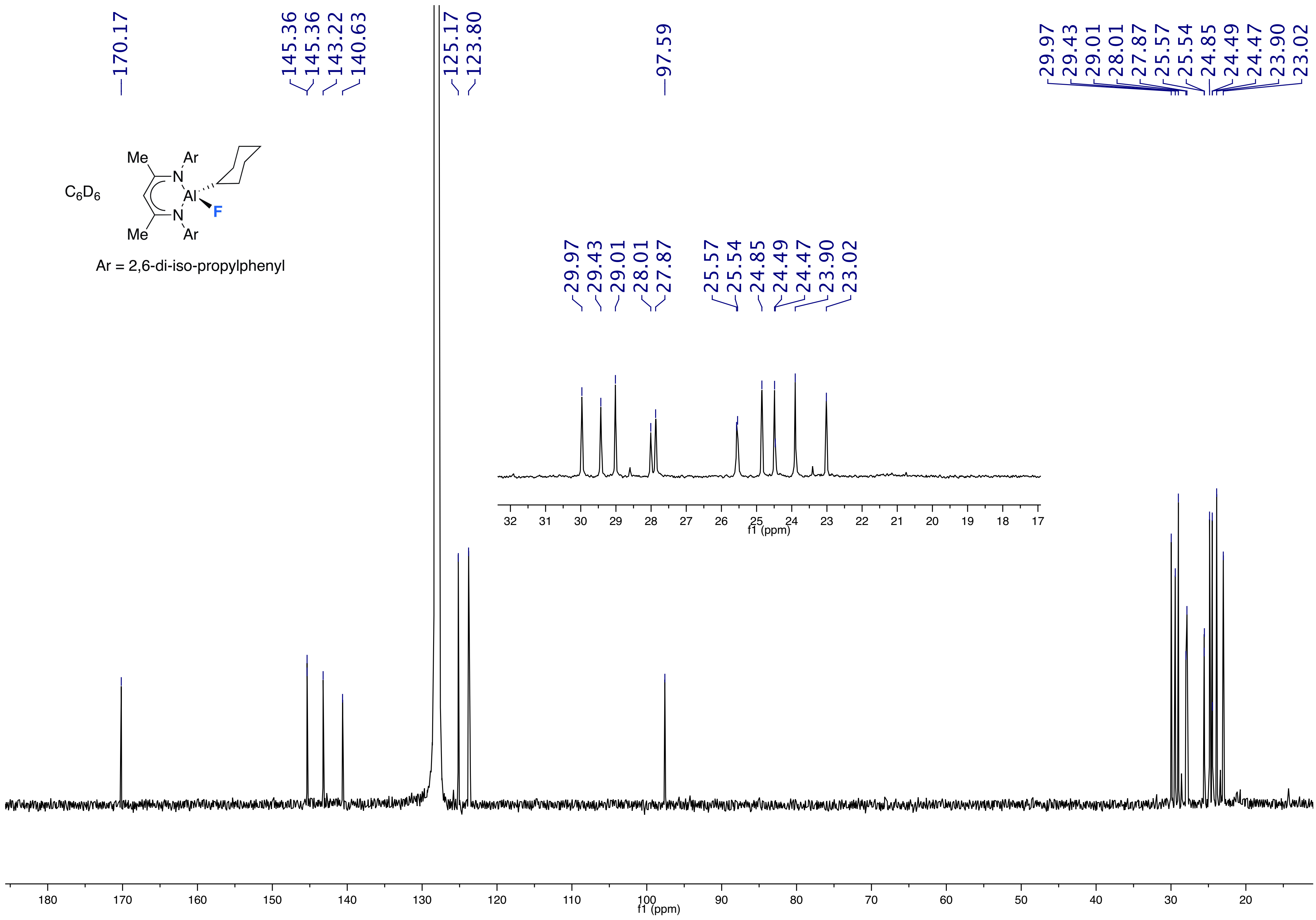

170.17
145.36
145.36
143.22
140.63
125.17
123.80
97.59
29.97
29.43
29.01
28.01
27.87
25.57
25.54
24.85
24.49
24.47
23.90
23.02
C6D6
Me
Ar
N
Al
F
Ar = 2,6-di-iso-propylphenyl
f1 (ppm)

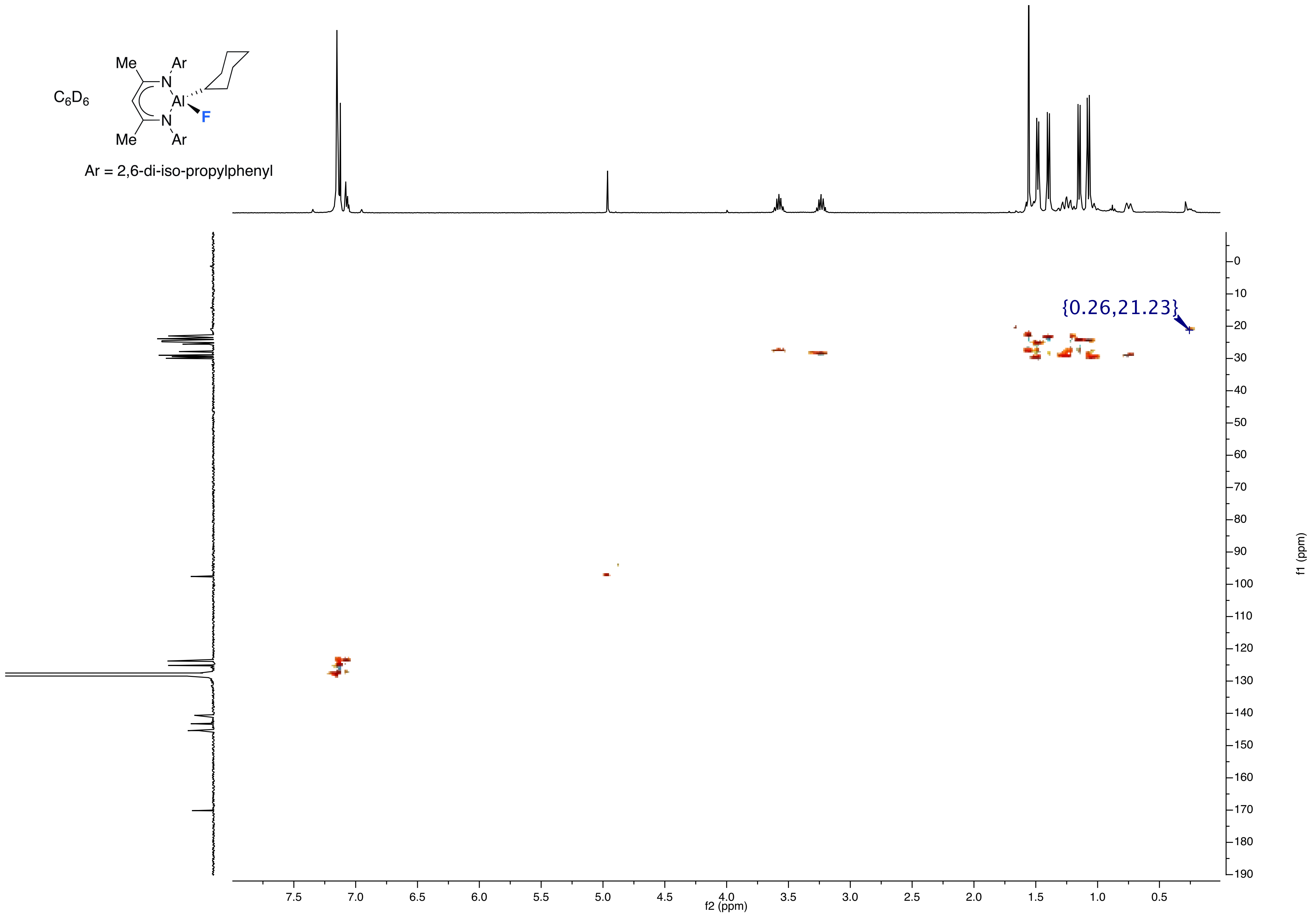

C6D6
Me
Ar
N
Al
F
N
Me
Ar
Ar = 2,6-di-iso-propylphenyl
{0.26,21.23}
f2 (ppm)
f1 (ppm)

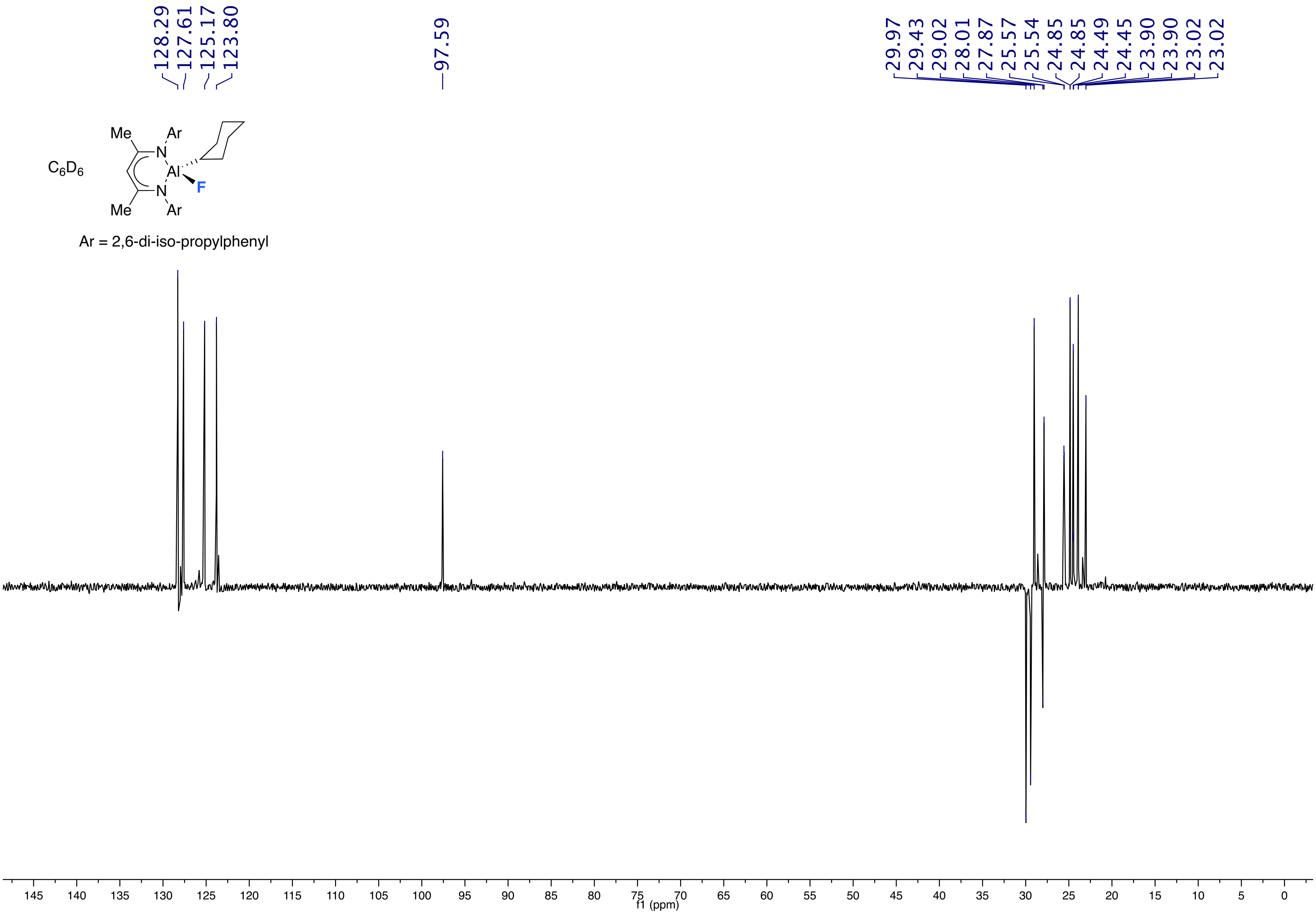

128.29
127.61
125.17
123.80
97.59
29.97
29.43
29.02
28.01
27.87
25.57
25.54
24.85
24.85
24.49
24.45
23.90
23.90
23.02
23.02
C6D6
Me
Ar
N
Al
F
N
Me
Ar
Ar = 2,6-di-iso-propylphenyl
145 140 135 130 125 120 115 110 105 100 95 90 85 80 75 70 65 60 55 50 45 40 35 30 25 20 15 10 5 0
f1 (ppm)

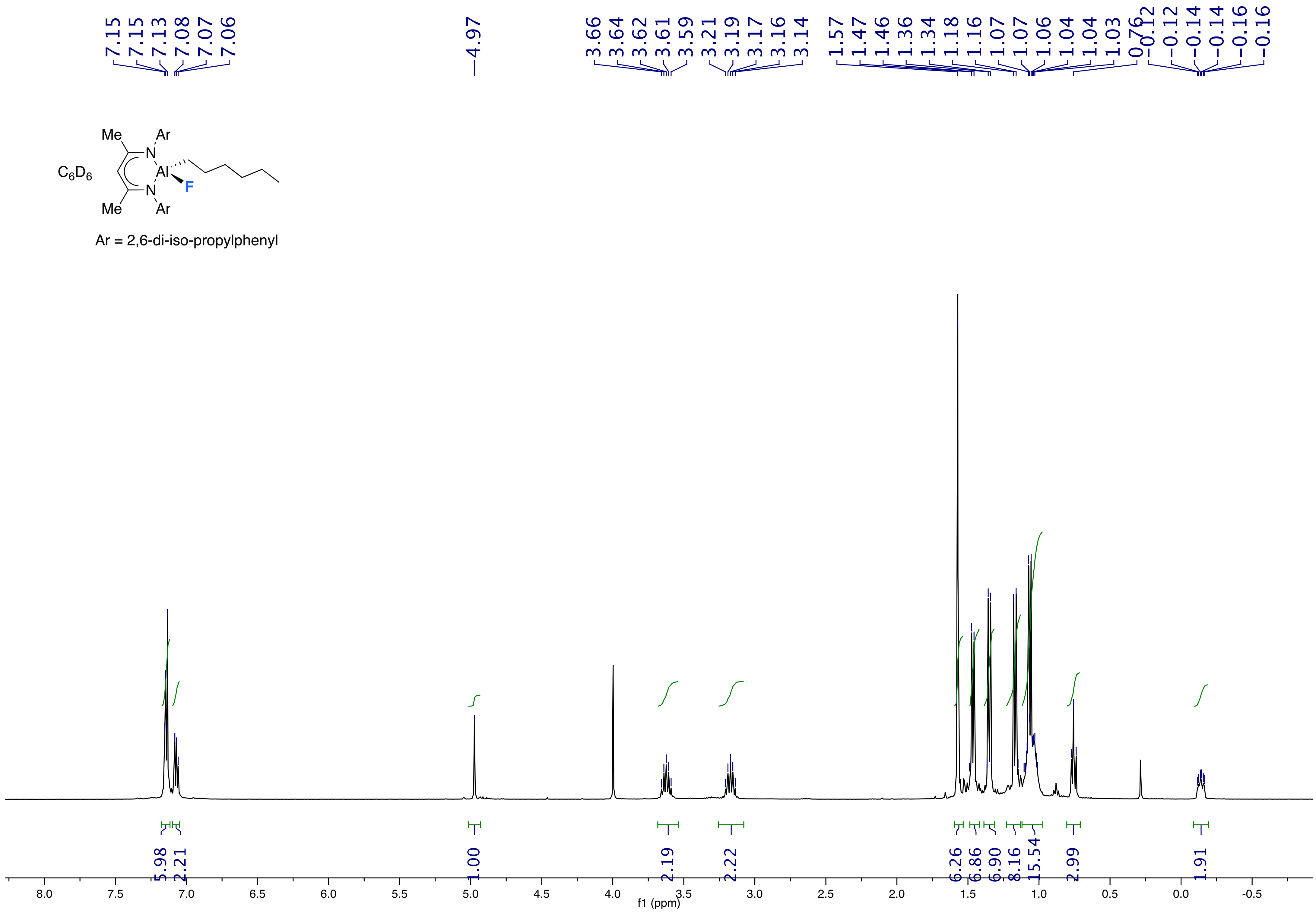

7.15 7.15 7.13 7.08 7.07 7.06
4.97
3.66 3.64 3.62 3.61 3.59 3.21 3.19 3.17 3.16 3.14
1.57 1.47 1.46 1.36 1.34 1.18 1.16 1.07 1.07 1.06 1.04 1.04 1.03 0.76
-0.12 -0.12 -0.14 -0.14 -0.16 -0.16
Me
Ar
N
Al
F
C6D6
Ar = 2,6-di-iso-propylphenyl
5.98 2.21 1.00 2.19 2.22 6.26 6.86 6.90 8.16 15.54 2.99 1.91
8.0 7.5 7.0 6.5 6.0 5.5 5.0 4.5 4.0 3.5 3.0 2.5 2.0 1.5 1.0 0.5 0.0 -0.5
f1 (ppm)

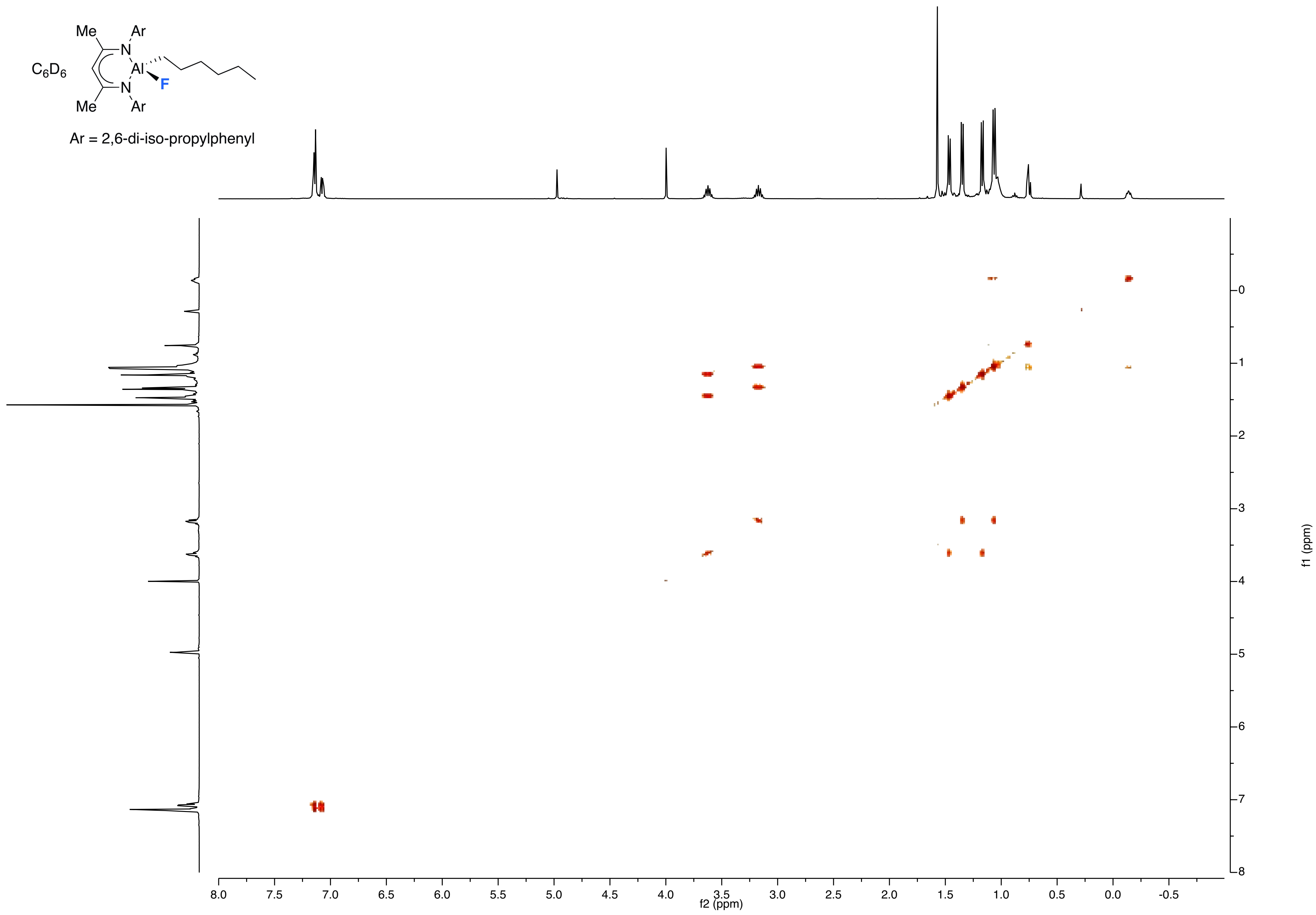

C6D6
Me
Ar
N
Al
F
Me
Ar
Ar = 2,6-di-iso-propylphenyl
f2 (ppm)
f1 (ppm)

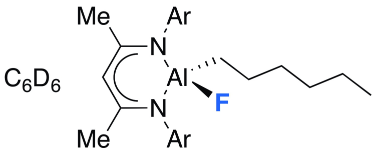

Ar = 2,6-di-iso-propylphenyl

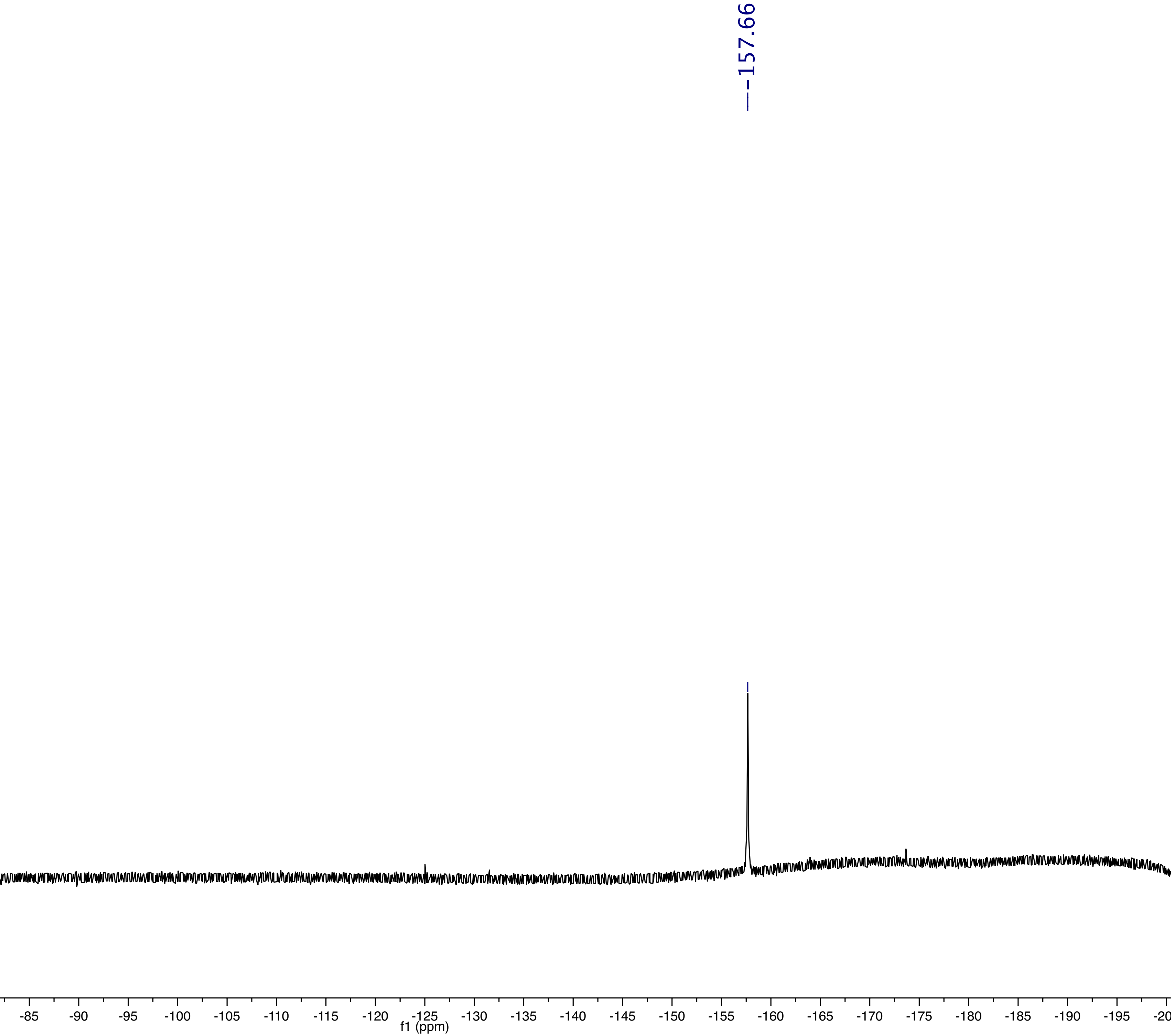

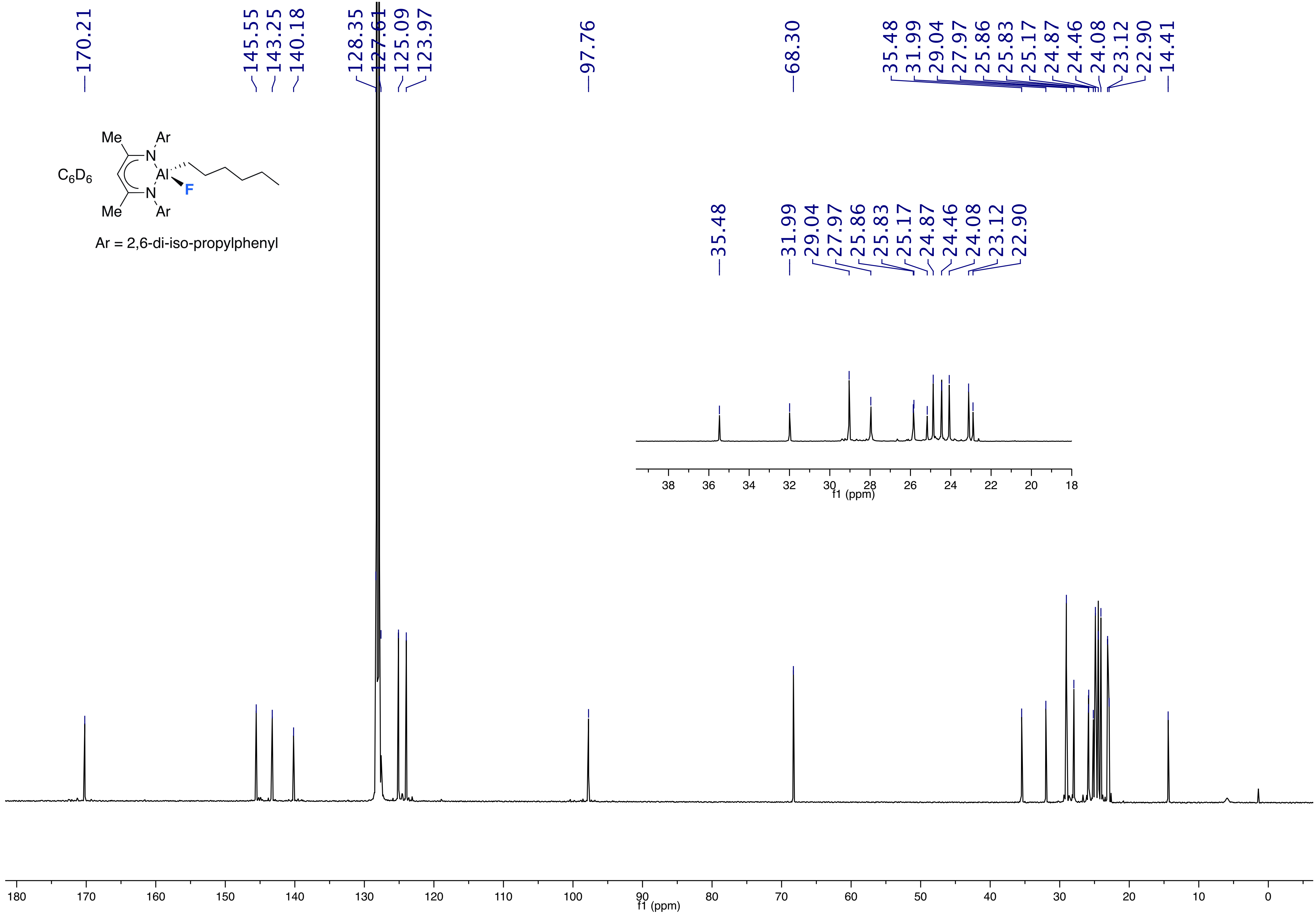

170.21
145.55
143.25
140.18
128.35
127.61
125.09
123.97
97.76
68.30
35.48
31.99
29.04
27.97
25.86
25.83
25.17
24.87
24.46
24.08
23.12
22.90
14.41
Me
Ar
N
Al
F
Me
Ar
C_6D_6
Ar = 2,6-di-iso-propylphenyl
f1 (ppm)

C_6D_6

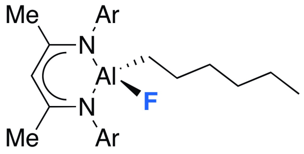

Ar = 2,6-di-iso-propylphenyl

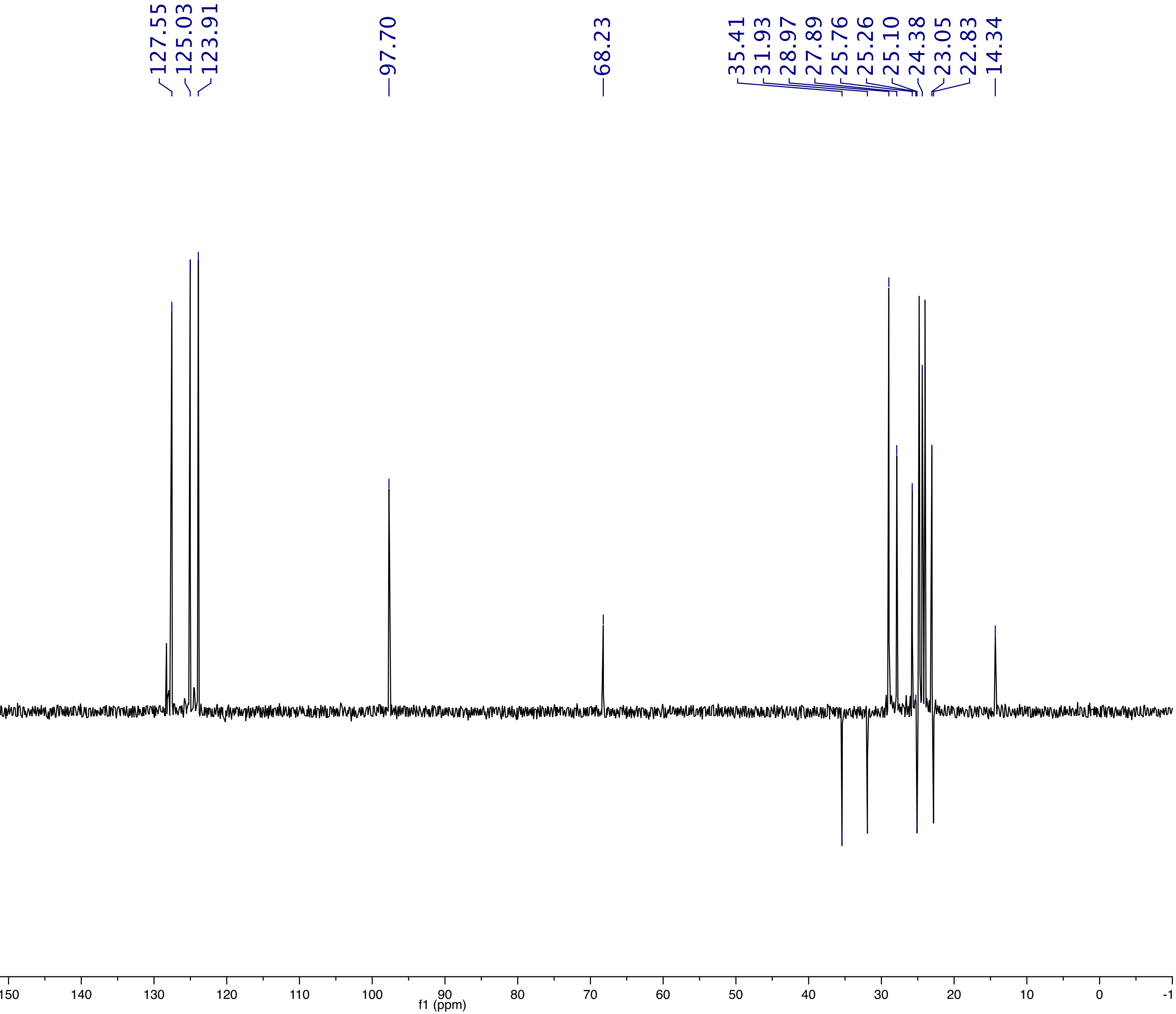

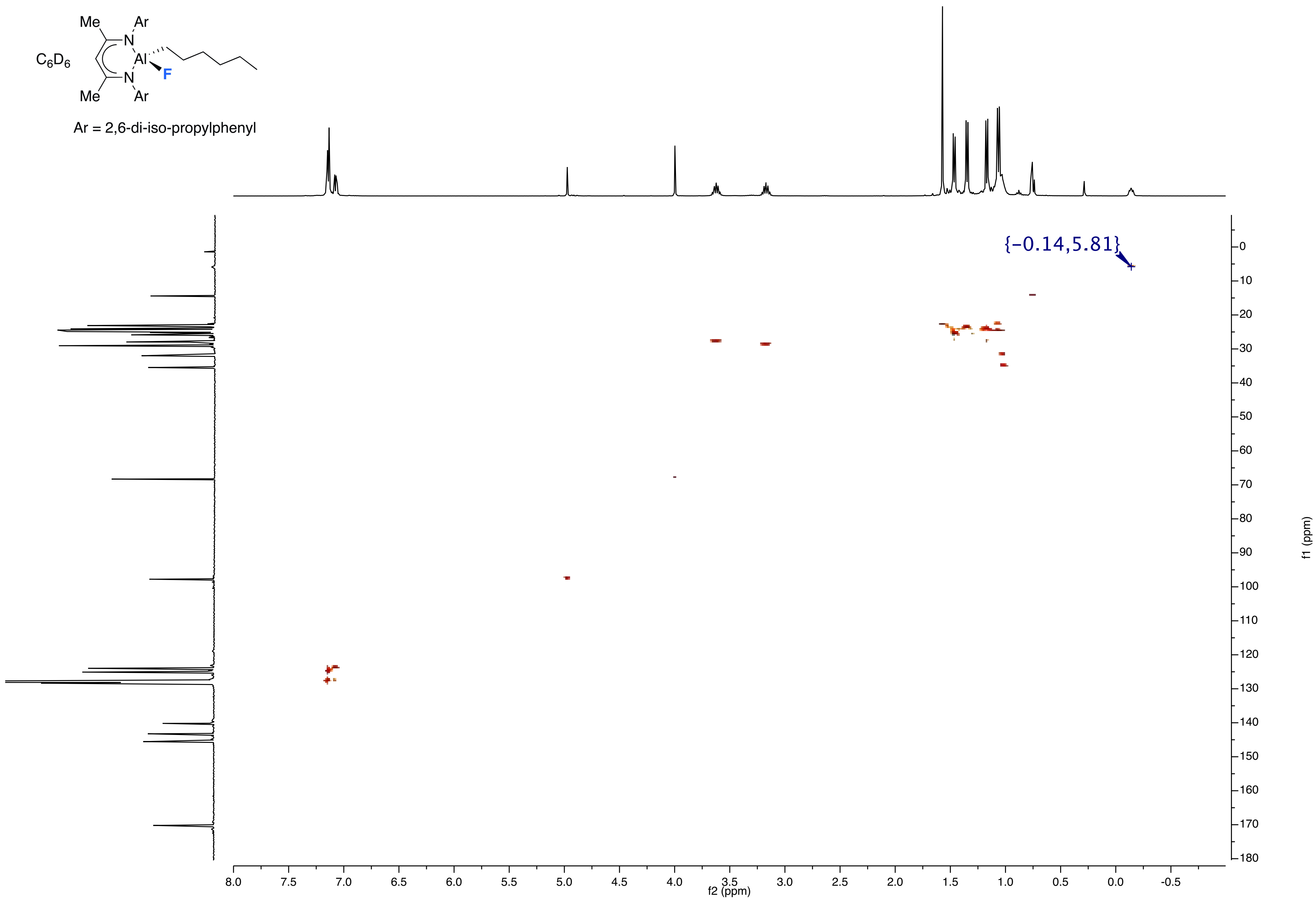
C6D6
Me
Ar
N
Al
F
Ar = 2,6-di-iso-propylphenyl
{-0.14,5.81}
f2 (ppm)
f1 (ppm)

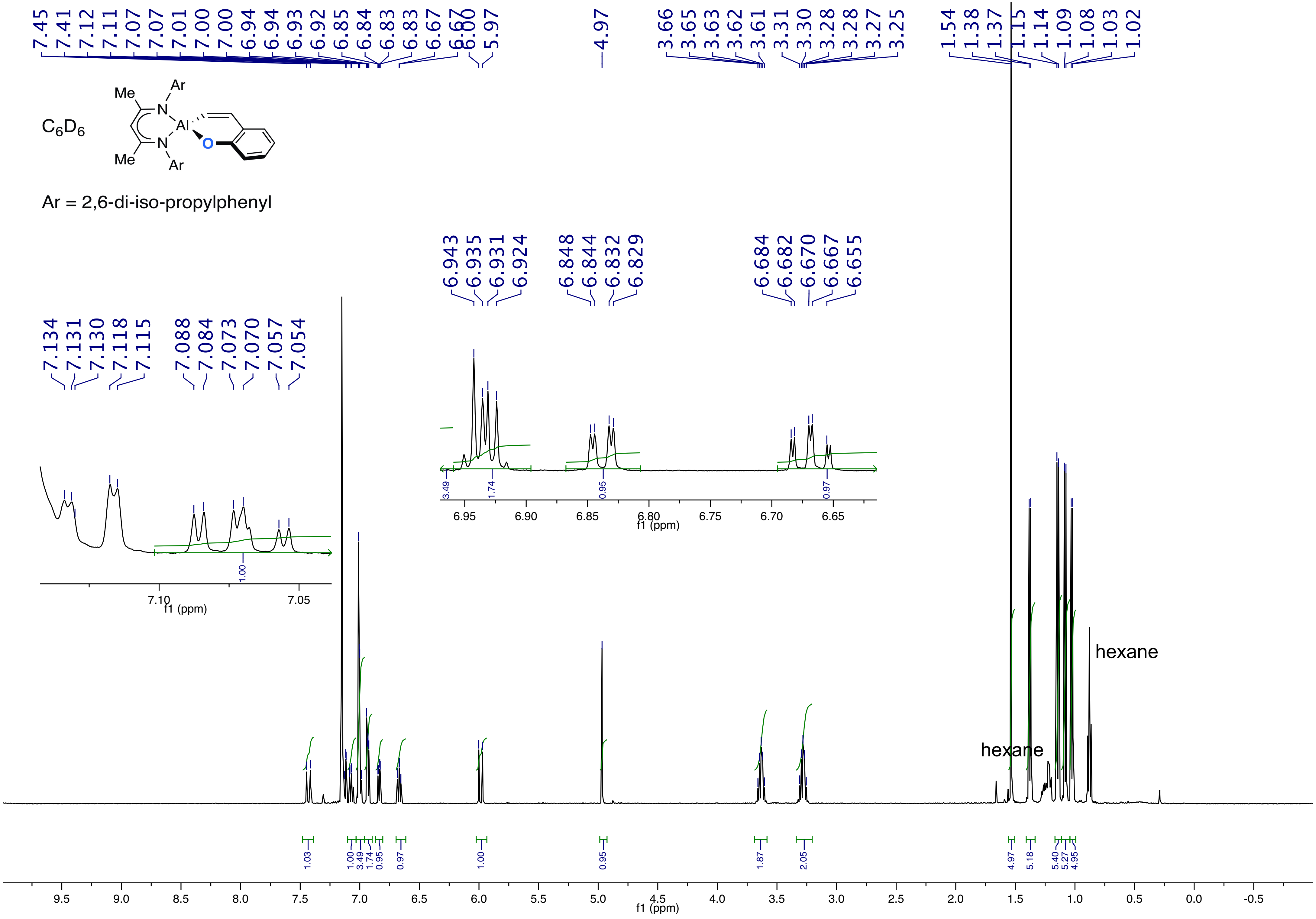

C6D6
Ar = 2,6-di-iso-propylphenyl
hexane
hexane
f1 (ppm)

C_6D_6

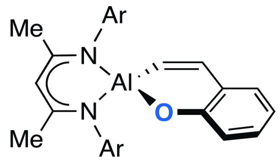

Ar = 2,6-di-iso-propylphenyl

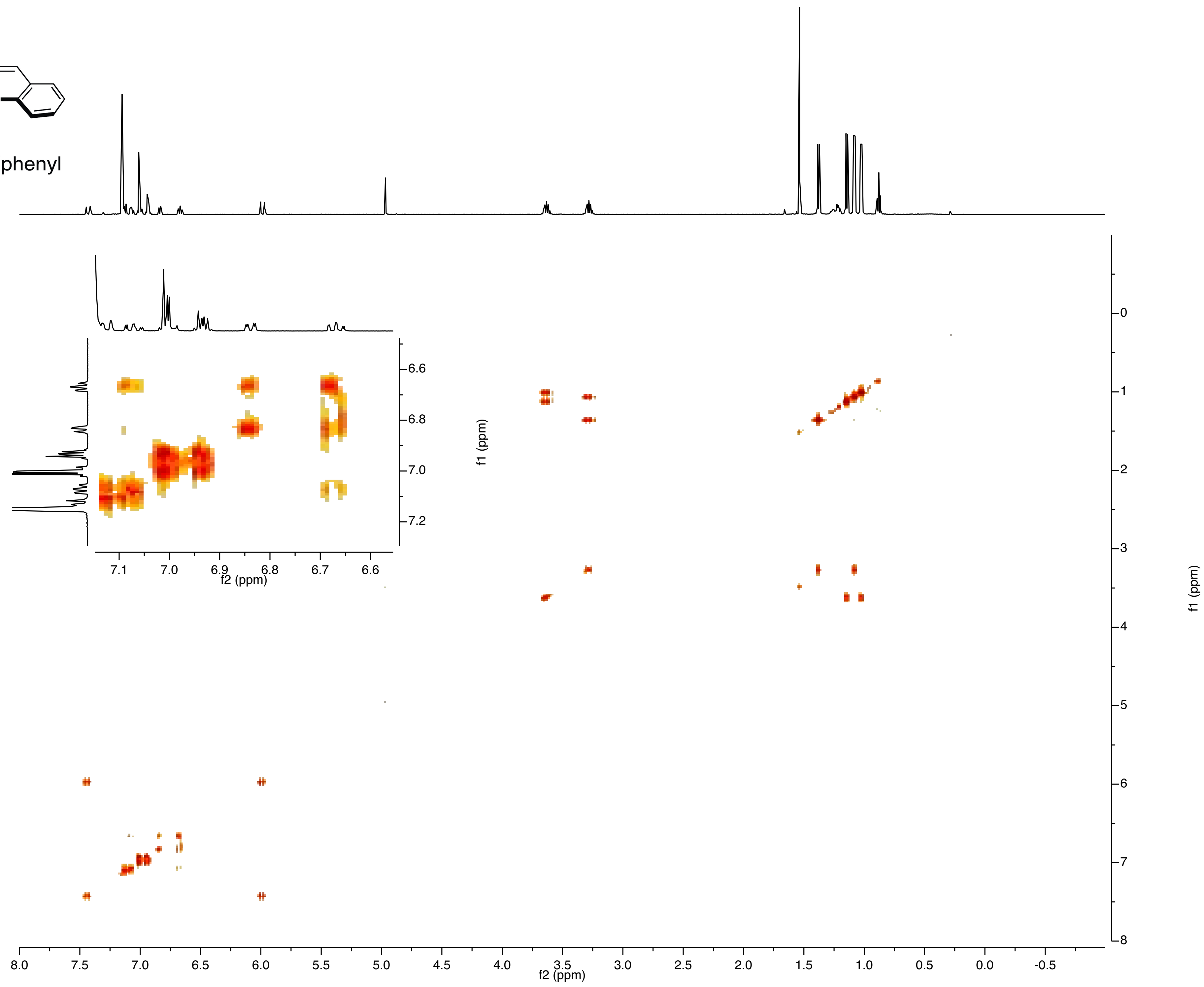

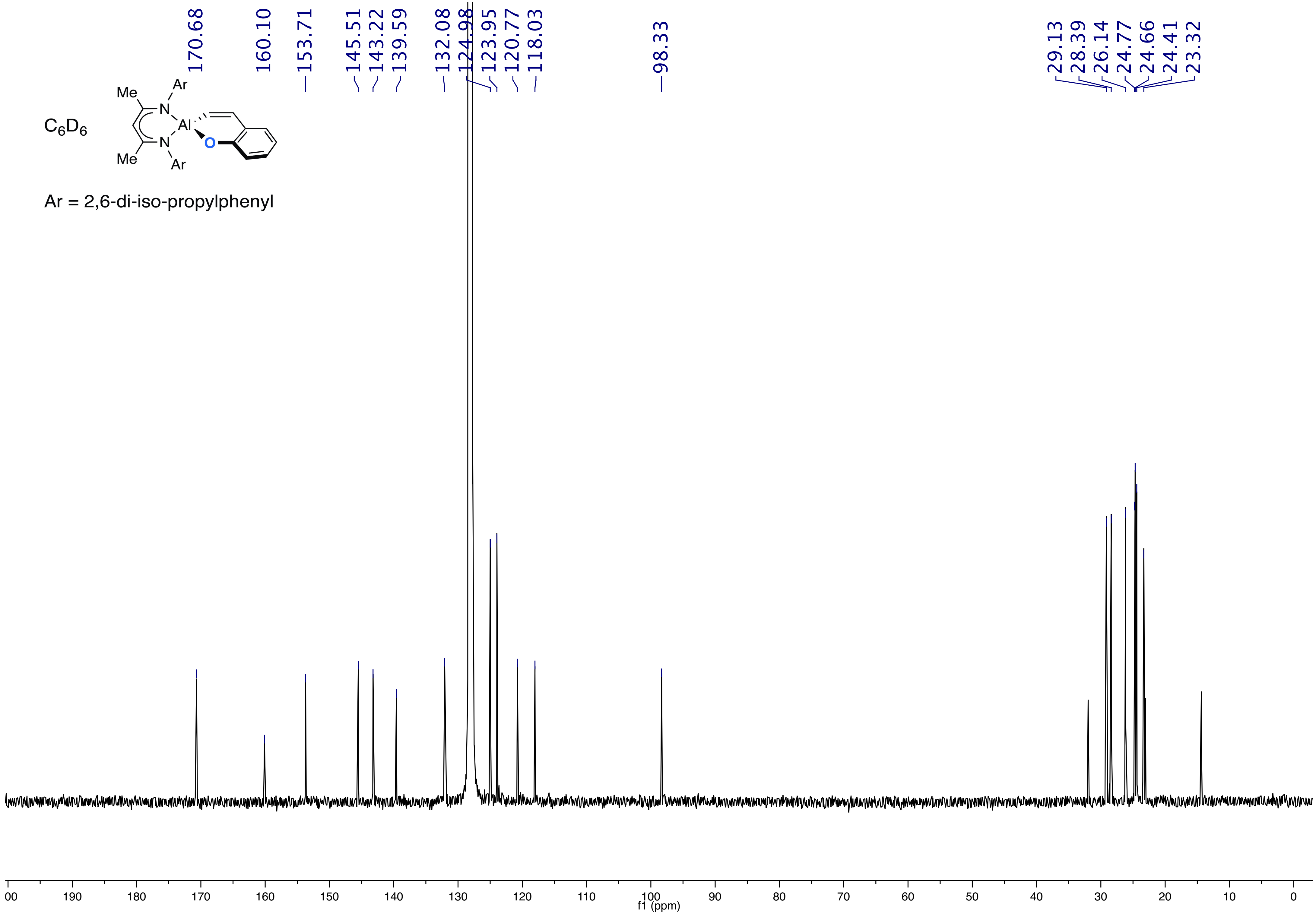

170.68
160.10
153.71
145.51
143.22
139.59
132.08
124.98
123.95
120.77
118.03
98.33
29.13
28.39
26.14
24.77
24.66
24.41
23.32
C_6D_6
Me
Ar
N
Al
N
O
Me
Ar
Ar = 2,6-di-iso-propylphenyl
00
190
180
170
160
150
140
130
120
110
100
90
80
70
60
50
40
30
20
10
0
f1 (ppm)

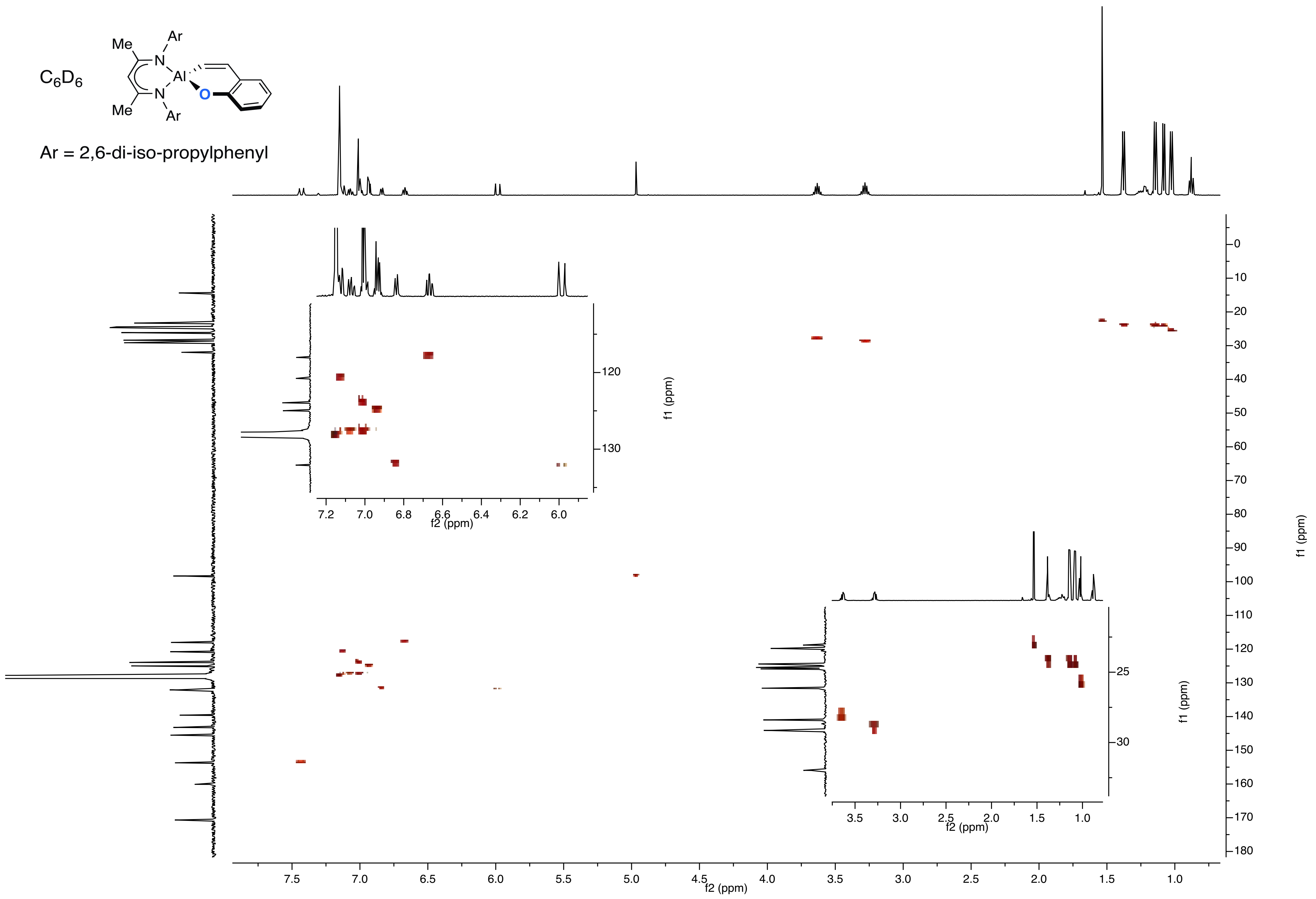

C6D6
Me
Ar
N
Al
O
Me
N
Ar
Ar = 2,6-di-iso-propylphenyl
f2 (ppm)
f1 (ppm)

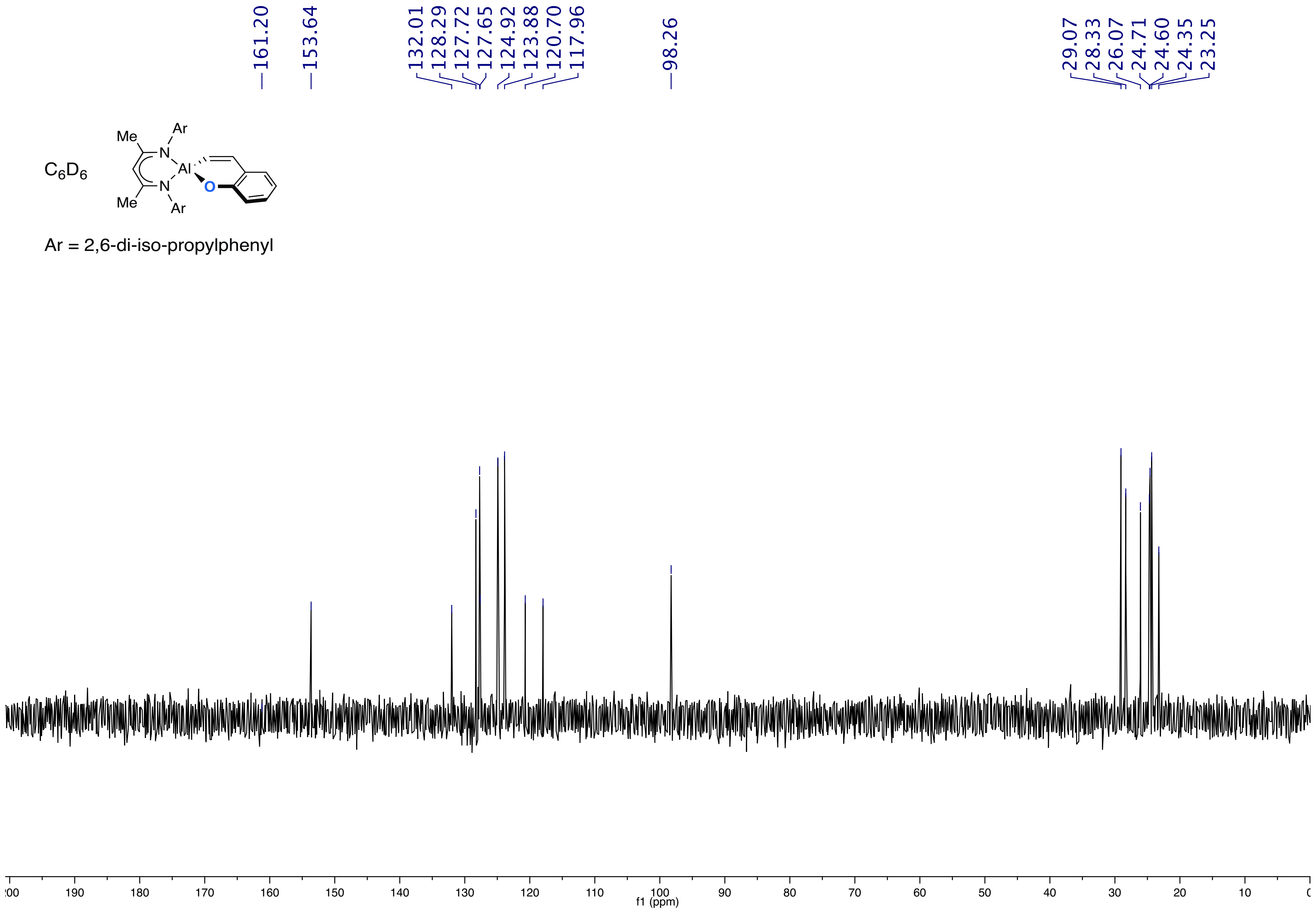
161.20
153.64
132.01
128.29
127.72
127.65
124.92
123.88
120.70
117.96
98.26
29.07
28.33
26.07
24.71
24.60
24.35
23.25
C6D6
Me
Ar
N
Al
O
Ar = 2,6-di-iso-propylphenyl
f1 (ppm)
